# Population genomics of rapidly invading lionfish in the Caribbean reveals signals of range expansion in the absence of spatial population structure

**DOI:** 10.1002/ece3.4952

**Published:** 2019-02-10

**Authors:** Eleanor K. Bors, Santiago Herrera, James A. Morris, Timothy M. Shank

**Affiliations:** ^1^ Biology Department Woods Hole Oceanographic Institution Woods Hole Massachusetts; ^2^ Marine Mammal Institute, Department of Fisheries and Wildlife Oregon State University Newport Oregon; ^3^ Department of Biological Sciences Lehigh University Bethlehem Pennsylvania; ^4^ National Oceanic and Atmospheric Administration, National Ocean Service National Centers for Coastal Ocean Science Beaufort North Carolina

**Keywords:** distributional shifts, genetic drift, invasive species, natural selection and contemporary evolution, RAD‐sequencing

## Abstract

Range expansions driven by global change and species invasions may have significant genomic, evolutionary, and ecological implications. During range expansions, strong genetic drift characterized by repeated founder events can result in decreased genetic diversity with increased distance from the center of the historic range, or the point of invasion. The invasion of the Indo‐Pacific lionfish, *Pterois volitans*, into waters off the US East Coast, Gulf of Mexico, and Caribbean Sea provides a natural system to study rapid range expansion in an invasive marine fish with high dispersal capabilities. We report results from 12,759 single nucleotide polymorphism loci sequenced by restriction enzyme‐associated DNA sequencing for nine *P. volitans *sampling areas in the invaded range, including Florida and other sites throughout the Caribbean, as well as mitochondrial control region D‐loop data. Analyses revealed low to no spatially explicit metapopulation genetic structure, which is partly consistent with previous finding of little structure within ocean basins, but partly divergent from initial reports of between‐basin structure. Genetic diversity, however, was not homogeneous across all sampled sites. Patterns of genetic diversity correlate with invasion pathway. Observed heterozygosity, averaged across all loci within a population, decreases with distance from Florida while expected heterozygosity is mostly constant in sampled populations, indicating population genetic disequilibrium correlated with distance from the point of invasion. Using an *F*_ST_ outlier analysis and a Bayesian environmental correlation analysis, we identified 256 and 616 loci, respectively, that could be experiencing selection or genetic drift. Of these, 24 loci were shared between the two methods.

## 1 INTRODUCTION

The distributions of species change over multiple temporal and spatial scales due to natural and human‐driven processes, such as glacial retreat over interglacial periods (Hewitt, 1999, 2000; Shum, Pampoulie, Kristinsson, & Mariani, 2015; Silva, Horne, & Castilho, 2014), global climate change (Harley et al., 2006; Parmesan & Yohe, 2003; Perry, 2005; Pinsky, Worm, Fogarty, Sarmiento, & Levin, 2013), local habitat alteration (Bradshaw et al., 2014), and non‐native species invasions (Lowry et al., 2013). Range expansions, a common type of distributional shift, can result in decreased genetic diversity with increasing distance from the center of the original range (Excoffier, Foll, & Petit, 2009), as has been observed in humans with distance from Africa (Ramachandran et al., 2005). Range expansion can also lead to evolution in life‐history traits (Phillips, Brown, & Shine, 2010). One genetic outcome of the spatial process of range expansion is allele surfing (alternatively called “gene surfing” or “mutation surfing”).

Allele surfing is a process by which an otherwise rare allele or new mutation rises to high frequency or fixation near a moving range margin because of repeated founder events through space and time (Edmonds, Lillie, & Cavalli‐Sforza, 2004; Hallatschek & Nelson, 2008; Klopfstein, 2005; Peischl, Dupanloup, Kirkpatrick, & Excoffier, 2013). The phenomenon of allele surfing is related to, but not exactly the same as, bottlenecks in diversity due to large founder events. Allele surfing can vary in strength, leaving either strong or subtle gradients in allele frequencies, and could potentially contribute to population genomic patterns during range expansion.

Invasive species are frequently studied in evolutionary biology as “natural experiments” or models to investigate the dynamics of invasion as well as adaptation to new environments (Barrett, 2015). Being able to predict the evolutionary dynamics of range expansion during invasion may be important for managing invasions and anticipating impacts of climate‐driven range shifts. Here, we use the invasion of the Indo‐Pacific lionfish, *Pterois volitans *[Linnaeus, 1758], as a model for rapid range expansion on a decadal time scale in a marine species with high dispersal capabilities.

**Figure 1:**
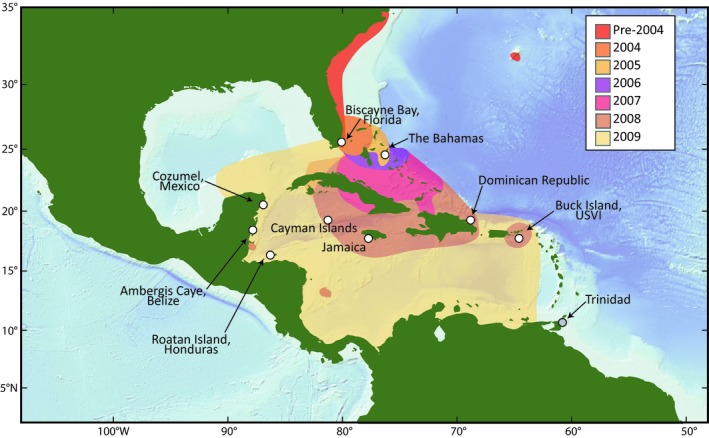
Map of the study region showing the nine sampling locations used for restriction enzyme‐associated DNA sequencing (samples from Trinidad, shown here with a gray circle, were used in mitochondrial analyses). Colored contours on the map indicate the extent of the invasion in the years from 2004 to 2009, by which point all of the nine sites had been invaded (see legend for dates)

The invasion of *P. volitans *and *Pterois miles* [Bennett, 1828] in the Western Atlantic and Caribbean Sea is unprecedented in both rate of geographic spread and ecological damage (Albins, 2015; Albins & Hixon, 2011; Hixon, Green, Albins, Akins, & Morris, 2016). First reported off Dania, Florida, in 1985, the lionfish invasion in the western Atlantic likely originated in southern Florida and has been characterized by a long incubation period and an immense expansion after establishment (Morris & Akins, 2009). In the late 1990s and early 2000s, lionfish began expanding northward; in 2004, they spread to the Bahamas; and in the years since, they have invaded the Caribbean Sea and the Gulf of Mexico (Ferreira et al., 2015; Schofield, 2009, 2010) (summarized in Figure 1). *Pterois volitans *is the most common species in the invasion, with *P. miles *mostly restricted to the northern part of the invaded range (Freshwater et al., 2009). While there has been speculation that *P. volitans *and *P. miles* could hybridize in the invaded range, Wilcox, Motomura, Matsunuma, and Bowen (2018) presented phylogenetic evidence that the lineage known as *P. volitans *in the invaded range may in fact represent a hybrid lineage between *P. miles* and another Pacific Ocean *Pterois* species. The present study focuses on the *P. volitans *lineage as it has historically been defined and uses that species name, while recognizing that there may be unresolved phylogenetic treatment of *Pterois*.

For lionfish in the invaded range, the majority of previous genetic studies have focused on mitochondrial sequencing to describe population genetic connectivity and population structure. To date, studies have identified just nine haplotypes of mitochondrial D‐loop in the invaded range but have not traced these directly back to a specific source in the native range, where genetic diversity is much greater (Betancur‐R et al., 2011; Butterfield et al., 2015; Freshwater et al., 2009; Johnson, Bird, Johnston, Fogg, & Hogan, 2016). While north‐to‐south (i.e., Western Atlantic to Caribbean) population differentiation has been reported in the invaded range, overall, a lack of metapopulation genetic structure has been reported within oceanic basins (Betancur‐R et al., 2011; Butterfield et al., 2015; Freshwater et al., 2009; Johnson et al., 2016; Toledo‐Hernández et al., 2014), with some local population structure reported in Puerto Rico (Toledo‐Hernández et al., 2014). Evidence of a bottleneck between the Caribbean and the Gulf of Mexico populations based on mitochondrial data has been presented based on the presence of only three of the four Caribbean D‐loop haplotypes in Gulf of Mexico populations (Johnson et al., 2016). Johnson et al. (2016) provide evidence for founder events when lionfish entered each new basin, potentially caused by gene flow restrictions related to oceanographic barriers. While these course‐scale patterns are congruent with large‐scale barriers to dispersal between oceanic basins that could have restricted gene flow into new areas as lionfish invaded, gradients in major allele frequency in populations across the lionfish's invasion pathway are beyond the scope of single locus mitochondrial studies.

The use of next‐generation sequencing (NGS) and other emerging genomic tools to provide novel insights into the evolutionary repercussions of range expansions and invasion dynamics is now widely recognized as the frontier in invasion genetics research, promising a synergy between previously intractable questions and burgeoning technologies (Barrett, 2015; Bock et al., 2015; Chown et al., 2014; Kirk, Dorn, & Mazzi, 2013; Rius, Bourne, Hornsby, & Chapman, 2015). Recent examples have demonstrated the power of NGS datasets to identify genomic regions undergoing neutral evolution and regions subject to natural selection with potential adaptive roles during an invasion (Tepolt & Palumbi, 2015; White, Perkins, Heckel, & Searle, 2013). In one such study, White et al. (2013) found evidence of both genetic drift and natural selection in populations of an invasive Bank Vole in Ireland, including patterns of decreased genetic diversity toward the moving range edge. In lionfish, genotype by sequencing (GBS) has been used to generate data for nuclear single nucleotide polymorphism (SNPs) in lionfish populations throughout Florida and the Gulf of Mexico, which is the most recently invaded body of water in that region (Pérez‐Portela et al., 2018). Using 1,220 SNPs generated with GBS, Pérez‐Portela report a lack of population structure between Florida and the Gulf of Mexico, contradicting previous reports from mitochondrial data regarding a founder event when the Gulf of Mexico was invaded.

This study presents genome‐wide SNP data for the invasive lionfish collected throughout the Caribbean, using 12,759 loci across nine populations. Data are analyzed from a range expansion perspective, not just a population structure perspective, identifying changes in genetic diversity with distance from the point of invasion. We test the hypothesis that lionfish range expansion is characterized by repeated founder effects through space and time at the moving range edge, leading to decreased genetic diversity (major allele frequency, allelic richness, and heterozygosity) with increased distance from the point of introduction in southeastern Florida. We predicted that signals of allele surfing would be detectable in the SNP data, in line with the theoretical predictions outlined above. Counter to these predictions, patterns of decreased diversity in the form of decreased major allele frequency or allelic richness were not observed in the data. However, decreases in average observed heterozygosity were observed, indicating higher levels of nonequilibrium population genetic dynamics near the range edge, with more central populations tending toward an equilibrium state. This may occur as populations become more established and the invasion front propagates forward. We also identify outlier loci in both Bayesian and *F*_ST_ analyses that may be under drift or under selection, potentially playing adaptive roles in certain lionfish populations.

## 2 METHODS

### 2.1 Sample collection

*Pterois volitans* individuals were collected from nine Caribbean sites for genomic analysis (Figure 1). Additional individuals were collected from Trinidad and included in mitochondrial analysis but not in restriction enzyme‐associated DNA sequencing (RAD‐seq) analysis. *Pterois volitans *specimens from Biscayne Bay, Florida, were collected by SCUBA divers from the US National Park Service in August and September of 2013 as part of ongoing collection programs. Fin clips were subsampled from each fish and stored in ethanol in a −20°C freezer. Similarly, samples from the US Virgin Islands were collected from Buck Island by divers from the University of the Virgin Islands between May of 2013 and February of 2014, and fin clips were subsampled. Samples from the Bahamas, the Dominican Republic, Jamaica, the Cayman Islands, Cozumel (Mexico), Belize, Honduras, and Trinidad were collected by divers throughout 2013, and tissue subsamples were archived in the US National Oceanic and Atmospheric Administration (NOAA) Beaufort Laboratory, Beaufort, North Carolina. Samples from Trinidad were only used in mitochondrial analyses due to DNA quality requirements for genomic analyses. All other sites were used for both mitochondrial and RAD‐sequencing analysis. Sections of muscle tissue from archived filets were subsampled at NOAA. Fish were identified to species when possible through meristics (i.e., morphological traits) at the collection site and later confirmed through molecular barcoding. If provided by collectors, latitude and longitude, depth, date of collection, sex, and standard or total length for each sample are given in Supporting Information Appendix S1. The latitude and longitude of the most common collection site per country were used in subsequent spatial analyses (Supporting Information Appendix S1). Tissue samples were shipped to the Woods Hole Oceanographic Institution in ethanol or frozen and then stored at −80°C until genomic DNA (gDNA) extraction.

To estimate the age of each sampled individual, and therefore the likely time of recruitment of the individuals used in this study, we calculated age from total length using a von Bertalanffy growth curve (Barbour, Allen, Frazer, & Sherman, 2011). For samples that lacked a standard length measurement but had a total length measurement, we utilized a conversion function to estimate standard length (Fogg et al., 2013). Distributions of estimated fish age and recruitment year are presented in Supporting Information Appendix S1.

### 2.2 DNA extraction, and mitochondrial DNA PCR, sequencing, and analysis

Genomic DNA was extracted from muscle or fin clip tissue using a CTAB and proteinase K digest, a phenol–chloroform purification, and an ethanol precipitation as described in Herrera, Watanabe, and Shank (2015b). gDNA was stored in AE buffer from a QIAGEN DNeasy Blood and Tissue Extraction Kit (Qiagen GmbH, Germany) at 4 or −20°C until gene amplification and sequencing.

Polymerase chain reactions (PCRs) were performed targeting the mitochondrial control region D‐loop with primers LionfishA‐H (5′‐CCATCTTAACATCTTCAG TG‐3′) and LionfishB‐L (5′‐CATATCAATATGATCTCAGTAC‐3′) (Freshwater et al., 2009). The thermocycler temperature profile consisted of 95° denaturing step for 3.5 min, then 30 cycles of 95° for 30 s, 51° for 45 s, 72° for 45 s, followed by a final extension step at 72° for 5 min. PCRs were purified using a QIAGEN PCR Purification Kit (Qiagen GmbH, Germany) and were sequenced in both directions using Sanger sequencing at Eurofins Operon Genomics (Eurofins MWG Operon LLC, Louisville, KY, USA). Sequences were edited and aligned using Geneious 8.1.5 (http://www.geneious.com, Kearse et al., 2012) and were compared to the previously published haplotypes. Mitochondrial sequence data were generated for a total of 217 individual *P. volitans* samples (23 from Florida, 17 from The Bahamas, 16 from the Dominican Republic, 25 from Jamaica, 15 from the Cayman Islands, 24 from Mexico, 18 from Belize, 24 from Honduras, 23 from the US Virgin Islands, and 32 from Trinidad). Genome‐wide SNP data were generated for a subset of 120 samples (Table 1; Table I.2 in Supporting Information Appendix S1).

**Table 1:** Population genomic summary statistics averaged over all loci and by population, as generated by the *Stacks*_*populations *program

Pop ID	*N*	*N* (*Stacks*)	Priivate	*P*	Obs Het	Exp Het	Pi	*F*_IS_
FLO	11	10.4786	421	0.9053	0.1177	0.1334	0.1402	0.0643
BAH	9	8.6021	620	0.9024	0.0997	0.139	0.1476	0.1304
CAY	11	10.0495	478	0.909	0.0718	0.1283	0.1351	0.1786
JAM	14	13.0478	746	0.9077	0.0873	0.1316	0.1369	0.1498
DOM	16	14.6043	656	0.907	0.0802	0.1325	0.1373	0.1779
MEX	7	6.5103	381	0.9096	0.0772	0.1261	0.1367	0.1474
BEL	20	17.7624	839	0.9057	0.0678	0.1338	0.1377	0.2272
HON	15	14.6673	781	0.9056	0.0882	0.1348	0.1397	0.1564
USV	16	13.7671	857	0.9069	0.0788	0.1329	0.1379	0.1828

Notes

Exp Het: expected heterozygosity; *F*_IS_: inbreeding coefficient; *N* (Stacks): average number of individuals used across all sampled loci; *N*: number of individuals sequenced; Obs Het: observed heterozygosity; *P*: major allele frequency (average); Pi: nucleotide diversity; private: number of private alleles in the population.

### 2.3 Restriction enzyme‐associated DNA sequencing

Restriction enzyme‐associated DNA sequencing library preparation using the *SbfI* restriction enzyme (restriction site: 5′‐CCTGCAGG‐3′) was carried out on concentration‐normalized gDNA by Floragenex Inc. (Eugene, OR, USA) in identical fashion to several other recent RAD‐seq studies (Herrera, Watanabe et al., 2015b; Reitzel, Herrera, Layden, Martindale, & Shank, 2013). gDNA was digested with the *SbfI* restriction enzyme, yielding fragments of many different lengths. Barcode tags (specific to each individual) 10 base pairs (bp) in length and an Illumina adaptor were ligated onto the sticky end of the cut site. Samples were then pooled, sheared, and size selected for optimal Illumina sequencing (Illumina Inc., San Diego, CA, USA). A subset of samples were prepared for paired‐end Illumina sequencing (Illumina Inc., San Diego, CA, USA) following the library prep protocol described in Baird et al. (2008), in order to generate longer sequencing assemblies for future analyses as well as provide possible comparisons of methods. For the preparation of the paired‐end sample library, a second adaptor was ligated to the second end of the read. All libraries were then enriched through PCR and sequenced by 96‐multiplex in a single lane of an Illumina Hi‐Seq 2000 sequencer (one lane for the single end sequencing, one for the paired end). For the samples sequenced in a paired‐end Illumina run, each sample was loaded twice to achieve a standard coverage (i.e., for one individual, two libraries were generated from two aliquots of gDNA with two barcodes).

### 2.4 RAD‐seq data processing and population genomic analyses

Using the *process_radtags* program in *Stacks *v1.19, raw Illumina, reads were filtered for quality with a minimum phred score of 10 in a sliding window of 15% read length (default settings) and sorted by individual‐specific barcode. Reads were truncated to 90 bp, including the 6‐bp restriction site. For the data generated with paired‐end sequencing, only the first read was used. Putative loci were generated using the *denovo_map.pl* pipeline in *Stacks* v1.35 (Catchen, Amores, Hohenlohe, Cresko, & Postlethwait, 2011) (references to *Stacks *from this point forward will all be to this version; please note that no changes were made to the *process_radtags *pipeline that would affect the interoperability of data between these two versions. This issue is further addressed in Supporting Information Appendix S2). We used a stack‐depth parameter (*‐m*) of 3, such that three reads were required to generate a stack (i.e., a locus); a within‐individual distance parameter (*‐M*) of 3, allowing for three SNP differences in a read; and a between‐individual distance parameter (‐*n*) of 3, allowing for three fixed differences between individuals to build a locus in the catalog. In initial exploratory analyses, altering the values of the within‐individual and between‐individual parameters did not significantly impact the number or identity of downstream loci called by *Stacks *(not reported).

Population summary statistics (allele frequencies, observed and expected heterozygosities, π, and *F*_IS_) were calculated by the *populations* program in *Stacks*, using loci found in eight of the nine populations and in at least 80% of individuals per population (command flags *‐p* 8, *‐r* 0.8). Information on the effect of changing the *‐p* and *‐r* flags is available in Supporting Information Appendix S2. For each RAD‐tag, only one SNP was used from 90‐bp sequence using the program flag *–write_random_snp* (if there were two or more SNPs in the sequence, *Stacks* would randomly choose one to analyze). Heterozygosity (observed and expected) values were also calculated in the R Package PopGenKit (R Core Team, 2016; https://cran.r-project.org/web/packages/PopGenKit/index.html) to provide secondary validations of reported values. Allelic richness was calculated using PopGenKit.

Three methods were used to describe the genetic structure of lionfish populations in the study area: principal component analysis (PCA), a *STRUCTURE* analysis, and *F*_ST_ calculations. The* smartpca* program in *EIGENSOFT* (Price et al., 2006) was used to perform a PCA of genetic diversity. Custom iPython notebooks used to convert *Stacks *PLINK output files into *EIGENSOFT* input files, and for the visualization of the PCA are available at the author's GitHub (https://github.com/ekbors/lionfish_pop_gen_scripts). *Smartpca *was run with four iterations of outlier removal (“*numoutlieriter” *= 4) with otherwise default parameters. In addition to the PCA,* fastSTRUCTURE* (Hubisz, Falush, Stephens, & Pritchard, 2009; Pritchard, Stephens, & Donnelly, 2000; Raj, Stephens, & Pritchard, 2014) was run with the number of genetic lineages (the value of *k*) set to values between one and 10 to assess genetic structure through a hierarchical analysis, and the program *chooseK.py* was run to select the value of *k* most consistent with the program's spatial structure model. *F*_ST_ values were calculated by the *populations* program in *Stacks *using a *p*‐value cutoff of 0.05 and a Bonferroni correction (using the “*bonferroni_gen” *flag in the *populations *program). In addition to these analyses, frequency spectra of the major alleles and of *F*_IS_ reported by *Stacks* were plotted in iPython. *F*_IS_ is calculated as $F_{\text{IS}}=\frac{H_{\text{S}}-H_{\text{I}}}{H_{\text{S}}}$ where *H*_S_ is the heterozygosity in the subpopulation and *H*_I _is the heterozygosity of the individual. The output from *Stacks* reports *F*_IS_ values of zero when the *H*_S_ is equal to zero (*p* = 1), but in these cases, the numerical value of *F*_IS_ is actually undefined. In order to remove these values, only *F*_IS_ values that were calculated when *H*_EXP_ > 0 were used.

Genetic diversity summary statistics were regressed against distance from the southern Florida collection site of Biscayne Bay using the *stats* package from *Scipy*, a collection of open source software for scientific computing (https://scipy.org). The least‐cost distance dispersal trajectories used in these regressions were calculated using the “gdistance” package in R with a bathymetric constraint from ETOPO1 (van Etten, 2015; R Core Team, 2016) with an additional requirement that pathways to sites to the west of Cuba first went around the east side of Cuba, a reasonable alteration considering the direction and strength of the Florida Current, as well as existing literature about the difficulty of dispersal of lionfish across that current (Johnston & Purkis, 2015). Other methods of measuring distance were explored, including Euclidian distance and a nonmodified least‐cost ocean distances (that did not require pathways to go around Cuba) that result in slightly different regressions but ultimately the same conclusions (Supporting Information Appendix S3).

In addition to the described approaches of regressing genetic diversity measurements with distance from Florida, we also implemented range expansion specific analyses (Peter & Slatkin, 2013). Using an R package developed by Peter and Slatkin (2013), we calculated *psi*, or the “directionality index,” which measures asymmetries in allele frequency data to evaluate the likely direction of expansion in a set of populations and the relative distance of a site to the center of the range (no prior definition of the origin of expansion is needed).

### 2.5 Blast2GO and locus identification

To annotate the RAD loci and infer possible links to gene function, we aligned the sequences to the nonredundant sequence database (restricted to teleost bony fishes) of National Center for Biotechnology Information (NCBI) using the BLASTx (Basic Local Alignment Search Tool) program as implemented in *Blast2GO *v2.5.1 (Conesa et al., 2005). We used an e‐value threshold of 1 × 10^−3^, a word size of three and a HSP length cutoff of 33. BLAST results were used to map Gene Ontology (GO) and annotate RAD loci.

### 2.6 Genome size estimation

To predict the size of the *P. volitans* genome based on the observed number of restriction sites (i.e., half the number of observed RAD loci), we used the linear model and parameter estimates for the *SbfI *enzyme described by Herrera, Reyes‐Herrera, and Shank (2015a) as implemented in the program *PredRAD* (Herrera, Reyes‐Herrera et al., 2015a). To generate a range for the number of restriction cut sites for *SbfI*, we ran the *Stacks *pipeline and *populations* program with several different permutations of parameters (Supporting Information Appendix S2) and then used a range of the number of total RAD loci generated by the different program runs.

### 2.7 Locus‐specific diversity analyses

Custom scripts were developed to identify groups of loci in the data with unique diversity patterns (https://github.com/ekbors/lionfish_pop_gen_scripts). Loci were identified for which (a) the major allele switched to the minor allele in at least one of the nine populations (i.e., “*p*” of the Hardy–Weinberg equation drops below 0.5); or (b) the difference between the maximum and minimum value of the overall major allele among the populations exceeded a defined value (measured at values of 0.5, 0.6, 0.7. 0.8, and 0.9). Loci identified by these filtering techniques were used in analyses of site frequency spectra (SFS) and *F*_IS_ to determine whether specific loci were driving and/or breaking patterns in the dataset, meaning that the forces driving those loci might be dominating the overall population data.

### 2.8 LOSITAN and BayEnv outlier analyses

To detect genomic outliers potentially under selection or strong genetic drift driven by expansion (which will yield similar diversity patterns), we used two analysis programs. *LOSITAN* (Antao, Lopes, Lopes, Beja‐Pereira, & Luikart, 2008) utilized data‐wide *F*_ST_ values to identify loci that were outliers in their *F*_ST_ values. We ran 1,000,000 simulations in *LOSITAN* for all nine populations with the options for “Forced mean *F*_ST_” and “Neutral *F*_ST_” selected. The false detection rate was set to 0.01, and a correction was implemented by the program.

The second program used for outlier analysis was* BayEnv 2.0* (Coop, Witonsky, Rienzo, & Pritchard, 2010; Günther & Coop, 2013), a program based on a Bayesian analysis that first develops a covariant matrix as a null model and then generates a linear model of relationship between diversity and an environmental factor. We used the calculated ocean distance from Florida as an environmental gradient against which to test patterns of diversity in the data. *BayEnv* controls for underlying population structure by generating a Bayes Factor for each locus indicating its relative goodness of fit to the linear model related to the environmental gradient. To interpret Bayes Factors, loci were binned in decimal intervals (randomly choosing *p* or *q* for each locus). Within each bin, each locus was ranked by Bayes Factor and that rank was divided by the number of loci in the bin. This created the empirical distribution from which loci in the top 5% and 1% of Bayes factor values were identified, as described in Coop et al. (2010) and Hancock et al. (2010).

Traditionally, these analyses are used to identify regions of the genome under selection. However, signals of allele surfing and strong genetic drift in the case of a range expansion could lead to allele frequency patterns correlating with distance or with expansion in ways that resemble the patterns of selection. As mentioned in the Introduction, these signals could vary in strength. Therefore, in some cases, the loci showing correlation to the gradient of distance may just as likely be the result of drift as selection (White et al., 2013).

## 3 RESULTS

### 3.1 No evidence of recent re‐introductions in mitochondrial data

Mitochondrial haplotypes consisting of 679 bp of the mitochondrial control D‐loop region were sequenced for 217 samples. Only five of the nine known haplotypes previously described were identified in these samples (Betancur‐R et al., 2011; Butterfield et al., 2015; Freshwater et al., 2009). These haplotypes correspond to previously named haplotypes H01, H02, H03, H04, and H06. Mitochondrial data do not indicate any new introductions of genetic material since the first publication of mitochondrial population genetic data in 2009 (Freshwater et al., 2009). Also in line with previous studies, distributional patterns and haplotype relationships largely corresponded to those described in Butterfield et al., 2015. For most locations, only two or three haplotypes were present in the tested sample, but all five haplotypes were found in the Bahamas samples. For a complete summary of the mitochondrial results, see Supporting Information Appendix S4.

### 3.2 High‐quality RAD‐seq and single nucleotide polymorphism data

Processing of raw Illumina data by the program *process_radtags* in *Stacks *resulted in the removal of <1% of the data due to poor sequencing quality, about 20% of the data due to ambiguity in the restriction site, and between 9% and 16% of reads due to ambiguous barcodes (inability to attribute a sequencing read to an individual). The number of reads removed varied slightly by sequencing type (single end vs. paired end) and by population (Supporting Information Appendix S2). The mean depth of reads for each individual, averaged over loci, was 24.5 reads, and the average of the standard deviations for each individual was 28.3. More in‐depth information on the depth of coverage is provided in the supplemental information (Supporting Information Appendix S2). The *Cstacks* program in *Stacks *generated a catalog of 1,376,469 putative loci, 12,759 of which were used by the *populations* program and in all subsequent analyses. The overall patterns of genetic diversity and genetic structure were not altered significantly in different parameter runs of *Stacks*. When more loci were included in analyses, heterozygosity increased overall—trends held the same shape but shifted upwards. This filtering‐diversity relationship is consistent with what is generally known about RAD‐sequencing approaches specifically under‐reporting diversity (Arnold, Corbett‐Detig, Hartl, & Bomblies, 2013) and being more conservative in the *populations* filtering for loci.

### 3.3 RAD‐Locus Identification

*Blast2GO *queries against all existing fish genome databases resulted in matches for 2,766 of the 12,759 loci (21.7%). In most cases, two RAD‐tag sequences matched to a BLAST result, which is consistent with having two “loci” sequenced in each direction away from the restriction site. These results could be used in concert with future draft and scaffold assemblies of the lionfish genome to confirm identity and location or RAD loci.

### 3.4 Genome size estimation and utilization

The number of RAD loci identified in multiple populations ranged from 9,502 to 48,079 with the majority of values between 30,000 and 50,000 (data are estimates from one catalog of loci generated by *denovomap.pl*, reviewed in Supporting Information Appendix S2). Given that sequencing in both directions from a cut site leads to two RAD “loci” at each cut site, we generated estimates for genome size for 15,000, 20,000, and 25,000 cut sites, representing the majority of putative values for cut sites (Table V.1 in Supporting Information Appendix S5). Estimates ranged from 370,725,631 to 680,784,288 bp. Considering these results, the 12,759 loci used in this study represent between 0.17% and 0.31% of the total lionfish genome.

### 3.5 Genomic diversity correlates with the invasion pathway

**Figure 2:**
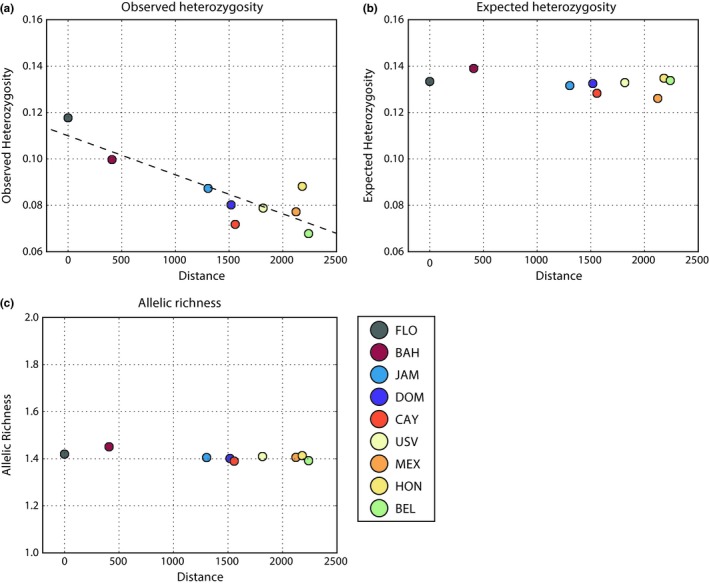
Summary statistics plotted against the “modified” ocean distance, measured from Florida. (a) Observed heterozygosity (*r* ^2^ = 0.744, *p*‐value = 0.003), (b) expected heterozygosity (no significant regression), and (c) allelic richness (no significant regression). BAH, The Bahamas; BEL, Belize; CAY, Cayman Islands; DOM, Dominican Republic; FLO, Florida; HON, Honduras; JAM, Jamaica; MEX, Mexico; USV, US Virgin Islands

Observed heterozygosity decreased linearly with distance from Florida (Figure 2a; Table 1) even though both allelic richness (average number of alleles per locus) and expected heterozygosity (calculated by *Stacks* as *2pq* from the Hardy–Weinberg equation) remained steady throughout the sampled range (Figure 2b,c). The difference between the expected and observed heterozygosity—a measure of deviation from Hardy–Weinberg equilibrium—increased with distance from Florida. All methods of measuring distance resulted in similar regressions for observed heterozygosity (Supporting Information Appendix S3). These range expansion patterns were observed despite a notable lack of spatial metapopulation genetic structure.

**Figure 3:**
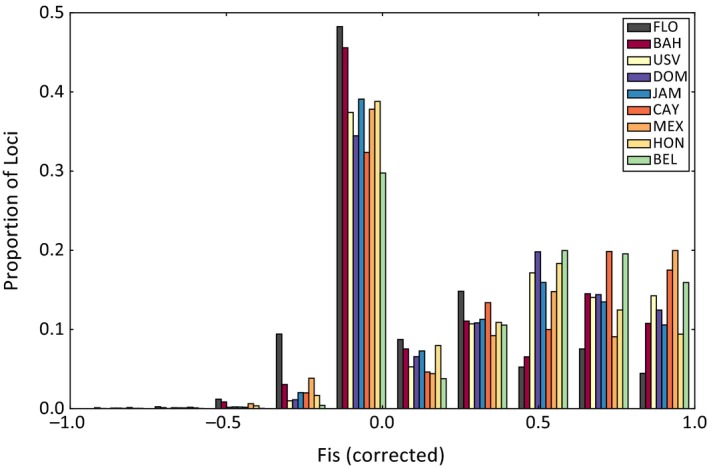
*F*_IS_ distributions showing the proportion of loci with *F*_IS_ values within each bin (number of bins = 10) for values between −1 and 1. Values of 0 reported by *Stacks* for loci for which the expected heterozygosity was 0 were removed from the data as described in the section 2. BAH, The Bahamas; BEL, Belize; CAY, Cayman Islands; DOM, Dominican Republic; FLO, Florida; HON, Honduras; JAM, Jamaica; MEX, Mexico; USV, US Virgin Islands

In general, SFS distributions for each population were similar to each other (Figure VI.1 in Supporting Information Appendix S6), with some subtle variation. Many evolutionary and population processes can affect the shape of a SFS distribution and it is difficult to discern what could be driving such subtle differences (Eldon, Birkner, Blath, & Freund, 2015). *F*_IS_ distributions in Florida and The Bahamas were closer to an equilibrium expectation of zero than *F*_IS_ distributions from populations closer to the moving range edge, which showed a thicker tail in the distribution skewing toward 1 (e.g., the Cayman Islands and Mexico; Figure 3).

### 3.6 Lack of Spatially explicit metapopulation genetic structure

**Figure 4:**
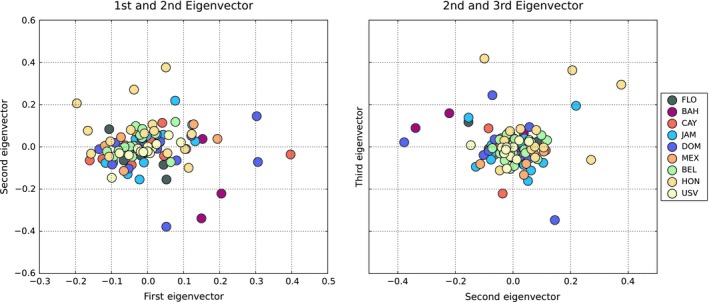
Principal component analysis generated by *smartpca* in *EIGENSOFT *here shown for the run with outliers removed. BAH, The Bahamas; BEL, Belize; CAY, Cayman Islands; DOM, Dominican Republic; FLO, Florida; HON, Honduras; JAM, Jamaica; MEX, Mexico; USV, US Virgin Islands

There was no obvious spatial metapopulation genetic structuring among the nine populations in the study region. PCA (Figure 4) revealed no clustering of defined populations with the first, second, and third components (e.g., eigenvectors) accounting for 11.03%, 10.44%, and 10.30%, respectively, of the variation in the dataset.

In order to determine the most likely number of genetic lineages (the value of *k*), or subpopulations, the *chooseK.py *program from* fastSTRUCTURE *was run for values of *k* between 1 and 10. The value of *k* that maximized marginal likelihood and that best explained the structure in the data (two different program metrics for assessing the appropriate value of *k*) was 1, indicating that the *fastSTRUCTURE* analysis fit the data best with just one genetic lineage. After a Bonferroni correction, many pairwise *F*_ST_ values calculated by *Stacks* were not statistically different from zero. For those that were, *F*_ST_ values showed very slight genetic differentiation among populations with significant values only for five pairings: Bahamas—Belize = 6.91 × 10^−5^; Caymans—Mexico = 1.1 × 10^−4^; Jamaica—Dominican Republic = 6.10 × 10^−5^; Jamaica—Honduras = 1.2 × 10^−4^; Dominican Republic—Honduras = 1.1 × 10^−^4. *F*_ST _values, therefore, do not reveal genetic differentiation of populations closer to the edge from those at the center of the range.

**Figure 5:**
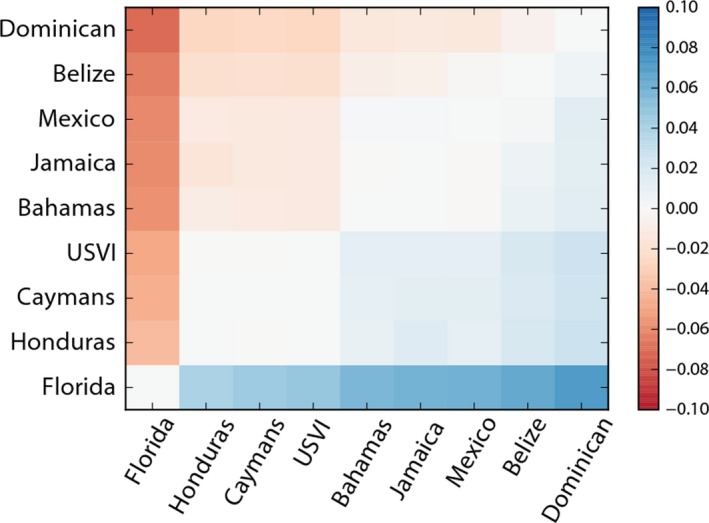
Directionality index heatmap. The directionality index, *psi, *measures asymmetries in allele frequencies. Here, values of *psi *have been arranged from lowest to highest—intended to parallel the ordering of sites from the closest to the origin of expansion to the furthest from the origin of expansion

The directionality index indicates another possible concept of distance from the point of invasion based on asymmetries of allele frequencies (Figure 5). The ordering of the index from lowest to highest indicates the “distance” in terms of the expansion from the center of the range. These data are ranked in the following order: Florida, Honduras, the Cayman Islands, US Virgin Islands, Jamaica, The Bahamas, Mexico, Belize, and the Dominican Republic. This order of distance, or invasion directionality is different from an expectation based solely on geographic proximity. Specifically, the results indicate that the Dominican Republic is more isolated from the center of the invasion than all other sites and that Honduras is much more connected to the core of the range even though it is geographically distant.

### 3.7 Locus‐specific patterns of spatial genetic diversity

There were 1,207 loci for which the value of *p*, or the major allele, defined as the allele most frequent across all the 120 samples, dropped below 0.5 in at least one population, meaning that for those loci, the major allele overall became the minor allele locally (called “Flip‐Flop loci” here; Table 2). There were 290 loci with a difference in the minimum and maximum allele frequency of at least 0.5, 55 with a difference of at least 0.6, 3 with a difference of at least 0.7, and 1 with a difference of at least 0.8. There were no loci with a minimum–maximum difference of 0.9 or greater. Of the loci that switched from major allele overall to minor allele in at least one population, 243 were also present in the 0.5 difference list. Therefore, 964 of the loci that switched between being a major and minor allele never had a maximum difference that exceeded 0.5. These loci are likely oscillating around a frequency of 0.5, not demonstrating dramatic changes throughout the invaded range. Such loci are sometimes attributed to balancing selection. The 243 loci with larger differences between their minimum and maximum values, however, could be driven by specific forces such as drift and directional selection.

**Table 2:** Comparisons of the loci identified as outliers by the two outlier analyses and loci identified through different filtering methods through custom analysis presented in this paper

Overlap of BayEnv top 5% with other filtering methods (total #loci = 616)					
	Lositan	Flip‐Flop	0.5 diff	0.6 diff	0.7 diff
Number shared	24	58	23	5	0

Overlap of Lositan Loci with other filtering methods (total #loci = 256)					
	BayEnv 1%	Flip‐Flop	0.5 diff	0.6 diff	0.7 diff
Number shared	5	100	118	43	3

Pairwise comparisons of major allele frequencies in populations closer to the central portions of the invaded range (“center populations” closer to the point of introduction, like Florida and The Bahamas) and those closer to the moving edge of the invaded range (“edge populations”) were used to detect specific loci for which frequencies were greater in the core of the invaded range than closer to the edge. From the list of loci that had a difference of 0.5 or more between maximum and minimum allele frequency, 115 had greater allele frequencies in Florida than in the USVI, 127 had greater allele frequencies in Florida than in Honduras, 106 had greater allele frequencies in the Bahamas than in the USV, and 122 had a greater allele frequency in the Bahamas than in Honduras. Additional pairwise comparison results showing counts of loci that overlap with different filtering requirements, including the outlier analyses described below are presented in Table 2.

### 3.8 Outlier loci

*LOSITAN *analyses identified 256 loci as possible targets of directional selection (having an *F*_ST_ outside the upper bound of the 95% confidence interval, with a correction for multiple tests). *BayEnv 2.0 *generated Bayes factors for the 12,759 analyzed loci. Taking the top 1% of loci from each bin captured 120 loci considered to have high enough Bayes Factors to be considered correlated to the linear regression model generated by the program *BayEnv*, taking the top 5% captured 616 loci. The top 5% of loci identified by *BayEnv* were then compared to the list of *F*_ST_ outliers generated by *LOSITAN* and were also compared to lists generated by the locus‐specific diversity analyses described above, including the loci with a change from major to minor allele, and those with large differences between their maximum and minimum frequencies (Table 2). Of the 615 loci in the top 5% of BayEnv analysis, 24 were also identified as outliers by *LOSITAN *analysis. Of these 24 loci, seven were putatively identified by Blast2GO. Several of these loci were identified by GO terms as being membrane proteins or involved in membranes (Table 3). SFS and *F*_IS_ for different subsets of alleles had different distributions (Figure SI.VI.2, Figure SI.VI.3 in Supporting Information Appendix S6).

**Table 3:** Blast2GO results for those loci that overlapped between the BayEnv top 5% and the Lositan outlier results

Locus	BLAST ID	GO terms
48803	“glutamate receptor NMDA 2B”	C:postsynaptic membrane; P:ion transmembrane transport; C:integral component of membrane; C:cell junction; P:ionotropic glutamate receptor signaling pathway; F:ionotropic glutamate receptor activity; F:extracellular glutamate‐gated ion channel activity
15012	“proto‐oncogene tyrosine kinase Src isoform X1”	F:ATP binding; P:peptidyl‐tyrosine phosphorylation; F:nonmembrane spanning protein tyrosine kinase activity; P:response to yeast
80176	“coiled‐coil domain‐containing KIAA1407 homolog”	No GO Terms
20821	“KN motif and ankyrin repeat domain‐containing 4‐like”	No GO Terms
11751	“membrane progestin receptor beta‐like”	C:integral component of membrane
54375	“CD209 antigen E isoform X2”	C:membrane
75133	“PREDICTED: uncharacterized protein LOC103354480”	No GO Terms

Notes

In the Gene Ontology (GO) terms, C: cellular component; F: molecular function; P: biological process.

Of the loci identified as outliers in both *BayEnv *and *LOSITAN* (Table 3), four that were associated with GO terms were more closely scrutinized. In a BLAST‐n query of the NCBI nucleotide database, only three of those four could be more specifically identified. These were, putatively, a glutamate receptor (locus 48803), a progestin receptor (locus 11751), and a tyrosine kinase (locus 15012; Supporting Information Appendix S7).

## 4 DISCUSSION

This study contains population genomic data generated using RAD‐seq for the invasive lionfish, *P. volitans,* collected from sites throughout the Caribbean Sea. Using 12,759 loci, we observed geographic patterns correlating diversity with distance from the point of invasion despite a lack of spatial metapopluation genetic structure. The most important of these patterns is the decrease of observed heterozygosity with distance from the point of invasion, despite no such pattern in expected heterozygosity, indicating a relationship between distance from the point of invasion and increased levels of disequilibrium. There are many factors that can lead to genetic disequilibrium, including essentially any violation of the Hardy–Weinberg assumptions. Considering the spatial relationship of the difference between observed and expected heterozygosity, the patterns of disequilibrium are likely related to spatial processes like expansion‐driven genetic drift or, as discussed below, other aspects of population dynamics during expansion.

No geographic metapopulation genetic structure was observed in either a PCA or *fastSTRUCTURE* analysis and only minor differences in *F*_ST_ values were observed across nine populations in the Caribbean Sea. While mitochondrial data were consistent with previous genetic investigations concluding that a strong initial bottleneck was followed by mixing and that Caribbean currents may have helped to produce low levels of population differentiation in lionfish (Butterfield et al., 2015); the RAD‐sequencing results for population structure did not find evidence of a genetic break between sites previously designated as Atlantic sites (Florida and The Bahamas) and those in the Caribbean (the rest of our study sites). The PCA and *F*_ST _analyses of RAD‐seq data presented here, rather, indicate that there is no structure. These findings are similar to those presented in Pérez‐Portela et al. (2018) for the Gulf of Mexico. Unfortunately, data reported in Pérez‐Portela et al. (2018) were generated using a different restriction enzyme than we used, precluding a direct side‐by‐side analysis of results. The results of both this study and Pérez‐Portela et al. (2018) indicate that it is possible that previously reported evidence of population structure among basins seen in mtDNA data could be solely driven by the absence of certain mitochondrial alleles in certain ocean basins. Pérez‐Portela et al. (2018) report results that demonstrate the same lack of structure in the Gulf of Mexico, which is likely connected to the greater Caribbean. While structure was not seen in PCA analyses, directionality index results did point to the possibility that certain locations are more or less connected to the rest of the invaded range. For example, results indicated that the Dominican Republic may be more isolated from the population in Florida than expected and that Honduras is more connected. While the effect of sample size and SNP filtering on directionality index needs to be investigated before solid inference can be made in our study area, these results could serve as the basis for hypotheses about how population structure may develop in Caribbean lionfish populations through time as the region moves closer to an equilibrium state.

Elevated *F*_IS_ values in populations farther from Florida could indicate cryptic structure (e.g., the Wahlund Effect, Hartl & Clark, 1997). Population densities closer to the edge of the invasion could be lower than those closer to the center of the invaded range, which could account for the observed signature of cryptic structure. These patterns in *F*_IS_ could be the result of smaller population size, which could be one force driving cryptic structure in the populations near the moving range boundary. While *F*_IS_ values are not specifically elevated in populations where fish were sampled from multiple reefs, it is also possible that reef patchiness in different locations, or other sources of habitat heterogeneity could contribute to differences in *F*_IS_. In three‐spined stickleback, elevated patterns of *F*_IS_ have been linked to cryptic structure in newly colonized freshwater populations (Catchen et al., 2013).

### 4.1 BLAST IDs of outlier loci

Using over 10,000 loci, we identified sites in the genome that break with equilibrium expectations. We identified 24 loci that are likely undergoing selection or strong genetic drift during expansion, including seven that were identified by BLAST, some of which were identified as membrane proteins by *Blast2GO *analysis. The majority of the identified outliers were not identified by BLAST. Because the knowledge of gene identity of our RAD‐tags can be only cursory, we are unable to specifically disentangle signals for beneficial or deleterious mutations or alleles; however, we are able to infer that any present signals of allele surfing are not present in enough loci to result in an overall signal in major allele frequencies averaged over the loci sequenced and compared among populations throughout the range. While beyond the scope of this work, future analysis of longer sequence reads from paired‐end RAD‐seq data could aid in better locating and identifying these loci in fish genomes (Bourgeois et al., 2013).

In analyzing selection outlier results, it is common practice to identify loci that are shared among multiple outlier identification software programs and consider them to be stronger candidates for selection than those found only by one program. This is done because it is widely acknowledged that each method has its own limitations and that biases in specific models or assumptions about selection may skew the data when only one program is used (Lotterhos & Whitlock, 2014). However, the *F*_IS_ and SFS for loci identified by *LOSITAN* and *BayEnv* result in visibly different genetic patterns (Supporting Information Appendix S6). *BayEnv* and *LOSITAN* use different metrics to find loci of interest, which look for “outliers” under different sets of expectations. LOSITAN identifies loci that have different allele frequencies (detected through deviations in *F*_ST_) in certain populations (Antao et al., 2008). *BayEnv*, in contrast, is based on detecting alleles that match a regression model with a certain parameter (in this paper, that parameter is distance from Florida), so it is likely to detect alleles with certain clines in the parameter space (Coop et al., 2010; Günther & Coop, 2013). While these results may not have specific biological relevance because they are in part driven by the nature of different outlier analyses, they serve as a reminder that finding loci that overlap among multiple outlier analyses may result in the loss of importance nuance about certain patterns that are demonstrated by different analysis methods.

Of the loci that were identified as outliers by both *BayEnv *and *LOSITAN *and further confirmed through more extensive BLAST analysis, three in particular stand out as being potentially important biologically for lionfish because of the functional roles of these proteins. These are (a) locus 48803, which is likely located in a glutamate receptor; (b) locus 11751, which is potentially part of a membrane progestin receptor sequence; and (c) locus 15012, which was identified as being located in a proto‐oncogene tyrosine kinase. These three loci could potentially be involved, respectively, in (a) learning and memory (Riedel, Platt, & Micheau, 2003); (b) gamete maturation (Tubbs, Pace, & Thomas, 2010), oocyte maturation (Zhu, Rice, Pang, Pace, & Thomas, 2003), and sperm hypermotility (Tan, Aizen, & Thomas, 2014); and (c) cell division and growth (Newsted & Giesy, 2000; Vivanco & Sawyers, 2002). These results present a starting point for further research into the role of these loci in lionfish biology and gene function. Further research into regions of the genome under selection in the lionfish's native range would be necessary to make any conclusions about the role of these loci in invasive lionfish evolution and adaptation.

### 4.2 Study design affects genetic signals

The year of recruitment of individual fish may affect genetic outcomes. For example, the fish sampled from the Cayman Islands—while collected in 2013—putatively recruited to the reef as early as 2005/2006, which would make them the oldest fish in the study (Supporting Information Appendix S1), which is interesting in light of the fact that observed heterozygosity of the Cayman Islands is lower than expected with the regression. This finding could be a result of the fact that the sampled Cayman fish are from an older age bracket, potentially representing a genetic cohort from earlier in the invasion. Fish sampled earlier could have lower diversity because they had just experienced the founder event characteristic of colonizing new locations and that population may quickly become more diverse after receiving more recruits from locations farther behind the advance invasion wave. Therefore, the age and recruitment date of samples in population genetic studies of range expansion could be important for understanding the dynamics of invasion genetics. Population genetic papers usually assume a sufficiently long time scale of genetic change that the specific age class of individuals sampled is unimportant to the genetic conclusions (Bors, Rowden, Maas, Clark, & Shank, 2012); however, in rapid range expansions when genetic change is expected, differences of 1 or 2 years could change the expected genetic signals. Caution, therefore, may be necessary when drawing conclusions from datasets that include samples from many time points throughout an invasion, a practice that common in several recent lionfish mitochondrial DNA population genetic analyses (Butterfield et al., 2015; Johnson et al., 2016).

This study is the first to employ RAD‐seq to explicitly describe range expansion genetics in lionfish across Caribbean sampling sites. While the development of NGS has accelerated the generation and analysis of large amounts of reduced representation genomic data, and the subsequent resolution of questions in the field of nonmodel species genomics (Merz et al., 2013; Reitzel et al., 2013; Therkildsen et al., 2013), analytical limitations due to the lack of whole genome sequences remain. For example, while the use of over 12,000 loci helped facilitate the identification of groups of loci that may be undergoing certain range expansion specific processes in this paper, it is possible that detecting specific clear examples of allele surfing (a rare allele becoming more common near a moving range boundary) may be difficult if only 0.31% of the genome is being sequenced (especially if, say, <1% of loci in the genome were demonstrating the pattern). So it is possible that allele surfing is still taking place and remain undetected by our analyses. The reduced representation strategy in this study samples less than one percent of the lionfish genome. Therefore, the likelihood of capturing loci that are experiencing allele surfing—unless there are many such loci—is low.

## 5 CONCLUSIONS AND IMPLICATIONS

Range expansions, while an undeniably important force in shaping genetic diversity across the planet, have limited signatures in some species due to the specific context of the expansion. Here, we have demonstrated that while not all the predicted patterns of expansion manifest themselves in the lionfish populations sampled, the range expansion process has led to disequilibrium closer to the range front. Populations of lionfish sampled in this study are well mixed and dispersal among sites is high, potentially precluding the detection of predicted decreases in allele frequency along the expansion axis. Genome‐level analyses revealed low to no spatially explicit metapopulation genetic structure, yet genetic diversity throughout the invaded range was not homogeneous. While patterns of genomic diversity correlated with invasion pathway, observed heterozygosity decreased with distance from Florida while expected heterozygosity remained mostly constant, indicating population genetic disequilibrium correlated with distance from the point of invasion.

Ultimately, the lack of decreases in major allele frequency or allelic richness across the invaded range suggests that the process of expansion is unlikely to cause long‐lasting limits to the adaptive potential of lionfish in their invaded range. It could also be inferred that signals of disequilibrium dissipate over time and space for the lionfish. Temporal comparisons of genetic diversity in a spatial context will be necessary to fully understand how a rapid invasion like that of the lionfish affects adaptive potential and the evolution of the species.

## CONFLICT OF INTEREST

None declared.

## AUTHOR CONTRIBUTIONS

EKB designed the research with assistance from TMS, JAM, and SH. EKB performed the research. EKB and SH completed the analyses. EKB wrote the manuscript with assistance from TMS, SH, and JAM.

## Data Availability

All custom scripts are available on the first author's GitHub page at https://github.com/ekbors/lionfish_scripts. Note that these contain Python code that was written as iPython Notebooks before changes to Jupyter. Demultiplexed RAD‐seq data are available on the GenBank SRA database (NCBI) under the bioproject accession number PRJNA510810. Information about the samples themselves is available in the Supplemental Materials section. All mitochondrial sequences generated matched existing sequences on GenBank uploaded from precious studies, meaning that no new sequences were not archived.

## References

[ece34952-bib-0001] Albins, M. A. (2015). Invasive Pacific lionfish *Pterois volitans* reduce abundance and species richness of native Bahamian coral‐reef fishes. Marine Ecology Progress Series, 522, 231–243. 10.3354/meps11159

[ece34952-bib-0002] Albins, M. A. , & Hixon, M. A. (2011). Worst case scenario: Potential long‐term effects of invasive predatory lionfish (*Pterois volitans*) on Atlantic and Caribbean coral‐reef communities. Environmental Biology of Fishes, 96, 1151–1157. 10.1007/s10641-011-9795-1

[ece34952-bib-0003] Antao, T. , Lopes, A. , Lopes, R. J. , Beja‐Pereira, A. , & Luikart, G. (2008). LOSITAN: A workbench to detect molecular adaptation based on a Fst‐outlier method. BMC Bioinformatics, 9, 323–325. 10.1186/1471-2105-9-323 18662398 PMC2515854

[ece34952-bib-0004] Arnold, B. , Corbett‐Detig, R. B. , Hartl, D. , & Bomblies, K. (2013). RADseq underestimates diversity and introduces genealogical biases due to nonrandom haplotype sampling. Molecular Ecology, 22, 3179–3190. 10.1111/mec.12276 23551379

[ece34952-bib-0005] Baird, N. A. , Etter, P. D. , Atwood, T. S. , Currey, M. C. , Shiver, A. L. , Lewis, Z. A. , … Johnson, E. A. (2008). Rapid SNP discovery and genetic mapping using sequenced RAD markers. PLoS ONE, 3, e3376 10.1371/journal.pone.0003376 18852878 PMC2557064

[ece34952-bib-0006] Barbour, A. B. , Allen, M. S. , Frazer, T. K. , & Sherman, K. D. (2011). Evaluating the potential efficacy of invasive lionfish (*Pterois volitans*) Removals. PLoS ONE, 6, e19666–e19667. 10.1371/journal.pone.0003376 21572951 PMC3091870

[ece34952-bib-0007] Barrett, S. C. H. (2015). Foundations of invasion genetics: The Baker and Stebbins legacy. Molecular Ecology, 24, 1927–1941. 10.1111/mec.13014 25442107

[ece34952-bib-0008] Betancur‐R, R. , Hines, A. , Acero, A. , Orti, G. , Wilbur, A. E. , & Freshwater, D. W. (2011). Reconstructing the lionfish invasion: Insights into Greater Caribbean biogeography. Journal of Biogeography, 38, 1281–1293. 10.1111/j.1365-2699.2011.02496.x

[ece34952-bib-0009] Bock, D. G. , Caseys, C. , Cousens, R. D. , Hahn, M. A. , Heredia, S. M. , Hübner, S. , … Rieseberg, L. H. (2015). What we still don't know about invasion genetics. Molecular Ecology, 24, 2277–2297. 10.1111/mec.13032 25474505

[ece34952-bib-0010] Bors, E. K. , Rowden, A. A. , Maas, E. W. , Clark, M. R. , & Shank, T. M. (2012). Patterns of deep‐sea genetic connectivity in the New Zealand region: Implications for management of benthic ecosystems. PLoS ONE, 7, e49474 10.1371/journal.pone.0049474 23185341 PMC3504039

[ece34952-bib-0011] Bourgeois, Y. X. C. , Lhuillier, E. , Cezard, T. , Bertrand, J. A. M. , Delahaie, B. , Cornuault, J. , … Thébaud, C. (2013). Mass production of SNP markers in a nonmodel passerine bird through RAD sequencing and contig mapping to the zebra finch genome. Molecular Ecology Resources, 13, 899–907. 10.1111/1755-0998.12137 23855484

[ece34952-bib-0012] Bradshaw, C. J. A. , Brook, B. W. , Delean, S. , Fordham, D. A. , Herrando‐Pérez, S. , Cassey, P. , … Araújo, M. B. (2014). Predictors of contraction and expansion of area of occupancy for British birds. Proceedings of the Royal Society B: Biological Sciences, 281, 20140744–20140744. 10.1098/rspb.2014.0744 PMC4046421 24827448

[ece34952-bib-0013] Butterfield, J. S. S. , Díaz‐Ferguson, E. , Silliman, B. R. , Saunder, J. W. , Buddo, D. , Mignucci‐Giannoni, A. A. , … Hunter, M. E. (2015). Wide‐ranging phylogeographic structure of invasive red lionfish in the Western Atlantic and Greater Caribbean. Marine Biology, 162, 773–781. 10.1007/s00227-015-2623-y

[ece34952-bib-0014] Catchen, J. M. , Amores, A. , Hohenlohe, P. , Cresko, W. , & Postlethwait, J. H. (2011). Stacks: Building and Genotyping Loci De Novo From Short‐Read Sequences. G3‐Genes Genomes Genetics, 1, 171–182. 10.1534/g3.111.000240 PMC3276136 22384329

[ece34952-bib-0015] Catchen, J. , Bassham, S. , Wilson, T. , Currey, M. , O'Brien, C. , Yeates, Q. , & Cresko, W. A. (2013). The population structure and recent colonization history of Oregon threespine stickleback determined using restriction‐site associated DNA‐sequencing. Molecular Ecology, 22, 2864–2883. 10.1111/mec.12330 23718143 PMC3712868

[ece34952-bib-0016] Chown, S. L. , Hodgins, K. A. , Griffin, P. C. , Oakeshott, J. G. , Byrne, M. , & Hoffmann, A. A. (2014). Biological invasions, climate change and genomics. Evolutionary Applications, 8, 23–46. 10.1111/eva.12234 25667601 PMC4310580

[ece34952-bib-0017] Conesa, A. , Götz, S. , Garcia‐Gomez, J. M. , Terol, J. , Talón, M. , & Robles, M. (2005). Blast2GO: A universal tool for annotation, visualization and analysis in functional genomics research. Bioinformatics, 21, 3674–3676. 10.1093/bioinformatics/bti610 16081474

[ece34952-bib-0018] Coop, G. , Witonsky, D. , Di Rienzo, A. , & Pritchard, J. K. (2010). Using environmental correlations to identify loci underlying local adaptation. Genetics, 185, 1411–1423. 10.1534/genetics.110.114819 20516501 PMC2927766

[ece34952-bib-0019] Edmonds, C. A. , Lillie, A. S. , & Cavalli‐Sforza, L. L. (2004). Mutations arising in the wave front of an expanding population. Proceedings of the National Academy of Sciences, 101, 975–979. https://doi.org/10.1073pnas.0308064100 10.1073/pnas.0308064100 PMC327127 14732681

[ece34952-bib-0020] Eldon, B. , Birkner, M. , Blath, J. , & Freund, F. (2015). Can the site‐frequency spectrum distinguish exponential population growth from multiple‐merger Coalescents? Genetics, 199(3), 841–856. 10.1534/genetics.114.173807 25575536 PMC4349076

[ece34952-bib-0021] Excoffier, L. , Foll, M. , & Petit, R. J. (2009). Genetic consequences of range expansions. Annual Review of Ecology, Evolution, and Systematics, 40, 481–501. 10.1146/annurev.ecolsys.39.110707.173414

[ece34952-bib-0022] Ferreira, C. E. L. , Luiz, O. J. , Floeter, S. R. , Lucena, M. B. , Barbosa, M. C. , Rocha, C. R. , & Rocha, L. A. (2015). First record of invasive lionfish (*Pterois volitans*) for the Brazilian coast. PLoS ONE, 10, e0123002–e123005. 10.1371/journal.pone.0123002 25901361 PMC4406615

[ece34952-bib-0023] Fogg, A. Q. , Hoffmayer, E. R. , Driggers, W. B. III , Campbell, M. D. , Pellegrin, G. J. , & Stein, W. (2013). Distribution and length frequency of invasive lionfish (*Pterois sp*.) in the northern Gulf of Mexico. Gulf and Caribbean Research, 25, 111–115. 10.18785/gcr.2501.08

[ece34952-bib-0024] Freshwater, D. W. , Hines, A. , Parham, S. , Wilbur, A. , Sabaoun, M. , Woodhead, J. , … Paris, C. B. (2009). Mitochondrial control region sequence analyses indicate dispersal from the US East Coast as the source of the invasive Indo‐Pacific lionfish Pterois volitans in the Bahamas. Marine Biology, 156, 1213–1221. 10.1007/s00227-009-1163-8

[ece34952-bib-0025] Günther, T. , & Coop, G. (2013). Robust identification of local adaptation from allele frequencies. Genetics, 195, 205–220. 10.1534/genetics.113.152462 23821598 PMC3761302

[ece34952-bib-0026] Hallatschek, O. , & Nelson, D. R. (2008). Gene surfing in expanding populations. Theoretical Population Biology, 73, 158–170. 10.1016/j.tpb.2007.08.008 17963807

[ece34952-bib-0027] Hancock, A. M. , Witonsky, D. B. , Ehler, E. , Alkorta‐Aranburu, G. , Beall, C. , Gebremedhin, A. , … Di Rienzo, A. (2010). Human adaptations to diet, subsistence, and ecoregion are due to subtle shifts in allele frequency. Proceedings of the National Academy of Sciences, 107, 8924–8930. 10.1073/pnas.0914625107 PMC3024024 20445095

[ece34952-bib-0028] Harley, C. D. G. , Hughes, A. R. , Hultgren, K. M. , Miner, B. G. , Sorte, C. J. B. , Thornber, C. S. , … Williams, S. L. (2006). The impacts of climate change in coastal marine systems. Ecology Letters, 9, 228–241. 10.1111/j.1461-0248.2005.00871.x 16958887

[ece34952-bib-0029] Hartl, D. L. , & Clark, G. C. (1997). Principles of population genetics. Sunderland: Sinauer Associates.

[ece34952-bib-0030] Herrera, S. , Reyes‐Herrera, P. H. , & Shank, T. M. (2015a). Predicting RAD‐seq Marker numbers across the eukaryotic tree of life. Genome Biology and Evolution, 7, 3207–3225. 10.1093/gbe/evv210 26537225 PMC4700943

[ece34952-bib-0031] Herrera, S. , Watanabe, H. , & Shank, T. M. (2015b). Evolutionary and biogeographical patterns of barnacles from deep‐sea hydrothermal vents. Molecular Ecology, 24, 673–689. 10.1111/mec.13054 25602032 PMC5006861

[ece34952-bib-0032] Hewitt, G. M. (1999). Post‐glacial re‐colonization of European biota. Biological Journal of the Linnean Society, 68, 87–112. 10.1111/j.1095-8312.1999.tb01160.x

[ece34952-bib-0033] Hewitt, G. M. (2000). The genetic legacy of the Quaternary ice ages. Nature, 405, 907–913. 10.1038/35016000 10879524

[ece34952-bib-0034] Hixon, M. A. , Green, S. J. , Albins, M. A. , Akins, J. L. , & Morris, J. A. Jr (2016). Lionfish: A major marine invasion. Marine Ecology Progress Series, 558, 161–165. 10.3354/meps11909

[ece34952-bib-0035] Hubisz, M. J. , Falush, D. , Stephens, M. , & Pritchard, J. K. (2009). Inferring weak population structure with the assistance of sample group information. Molecular Ecology Resources, 9, 1322–1332. 10.1111/j.1755-0998.2009.02591.x 21564903 PMC3518025

[ece34952-bib-0036] Johnson, J. , Bird, C. E. , Johnston, M. A. , Fogg, A. Q. , & Hogan, J. D. (2016). Regional genetic structure and genetic founder effects in the invasive lionfish: Comparing the Gulf of Mexico, Caribbean and North Atlantic. Marine Biology, 163, 1–7. 10.1007/s00227-016-2981-0

[ece34952-bib-0037] Johnston, M. W. , & Purkis, S. J. (2015). Hurricanes accelerated the Florida‐Bahamas lionfish invasion. Global Change Biology, 21, 2249–2260. 10.1111/gcb.12874 25620639

[ece34952-bib-0038] Kearse, M. , Moir, R. , Wilson, A. , Stones‐Havas, S. , Cheung, M. , Sturrock, S. , … Drummond, A. (2012). Geneious Basic: An integrated and extendable desktop software platform for the organization and analysis of sequence data. Bioinformatics, 28, 1647–1649. 10.1093/bioinformatics/bts/199 22543367 PMC3371832

[ece34952-bib-0039] Kirk, H. , Dorn, S. , & Mazzi, D. (2013). Molecular genetics and genomics generate new insights into invertebrate pest invasions. Evolutionary Applications, 6, 842–856. 10.1111/eva.12071 29387170 PMC5779122

[ece34952-bib-0040] Klopfstein, S. (2005). The fate of mutations surfing on the wave of a range expansion. Molecular Biology and Evolution, 23, 482–490. 10.1093/molbev/msj057 16280540

[ece34952-bib-0041] Lotterhos, K. E. , & Whitlock, M. C. (2014). Evaluation of demographic history and neutral parameterization on the performance of FST outlier tests. Molecular Ecology, 23, 2178–2192. 10.1111/mec.12725 24655127 PMC4228763

[ece34952-bib-0042] Lowry, E. , Rollinson, E. J. , Laybourn, A. J. , Scott, T. E. , Aiello‐Lammens, M. E. , Gray, S. M. , … Gurevitch, J. (2013). Biological invasions: A field synopsis, systematic review, and database of the literature. Ecology and Evolution, 3, 182–196. 10.1002/ece3.431 PMC3568853 23404636

[ece34952-bib-0043] Merz, C. , Catchen, J. M. , Hanson‐Smith, V. , Emerson, K. J. , Bradshaw, W. E. , & Holzapfel, C. M. (2013). Replicate phylogenies and post‐glacial range expansion of the pitcher‐plant mosquito, Wyeomyia smithii, in North America (WJ Etges, Ed,). PLoS ONE, 8, e72262–e72268. 10.1371/journal.pone.0072262 24039746 PMC3765167

[ece34952-bib-0044] Morris, J. A. Jr , & Akins, J. L. (2009). Feeding ecology of invasive lionfish (Pterois volitans) in the Bahamian archipelago. Environmental Biology of Fishes, 86, 389–398. 10.1007/s10641-009-9538-8

[ece34952-bib-0045] Newsted, J. L. , & Giesy, J. P. (2000). Epidermal growth factor receptor‐protein kinase interactions in hepatic membranes of rainbow trout (Oncorhynchus mykiss). Fish Physiology and Biochemistry, 22, 181–189.

[ece34952-bib-0046] Parmesan, C. , & Yohe, G. (2003). A globally coherent fingerprint of climate change impacts across natural systems. Nature, 421, 37–42. 10.1038/nature01286 12511946

[ece34952-bib-0047] Peischl, S. , Dupanloup, I. , Kirkpatrick, M. , & Excoffier, L. (2013). On the accumulation of deleterious mutations during range expansions. Molecular Ecology, 22, 5972–5982. 10.1111/mec.12524 24102784

[ece34952-bib-0048] Pérez‐Portela, R. , Bumford, A. , Coffman, B. , Wedelich, S. , Davenport, M. , Fogg, A. , … Oleksiak, M. F. (2018). Genetic homogeneity of the invasive lionfish across the Northwestern Atlantic and the Gulf of Mexico based on single nucleotide polymorphisms. Scientific Reports, 8, 5062 10.1038/s41598-018-23339-w 29567984 PMC5864727

[ece34952-bib-0049] Perry, A. L. (2005). Climate change and distribution shifts in marine fishes. Science, 308, 1912–1915. 10.1126/science.1111322 15890845

[ece34952-bib-0050] Peter, B. M. , & Slatkin, M. (2013). Detecting range expansions from genetic data. Evolution, 67, 3274–3289. 10.1111/evo.12202 24152007 PMC4282923

[ece34952-bib-0051] Phillips, B. L. , Brown, G. P. , & Shine, R. (2010). Life‐history evolution in range‐shifting populations. Ecology, 91, 1617–1627. 10.1890/09-0910.1 20583704

[ece34952-bib-0052] Pinsky, M. L. , Worm, B. , Fogarty, M. J. , Sarmiento, J. L. , & Levin, S. A. (2013). Marine taxa track local climate velocities. Science, 341, 1239–1242. 10.1126/science.1239352 24031017

[ece34952-bib-0053] Price, A. L. , Patterson, N. J. , Plenge, R. M. , Weinblatt, M. E. , Shadick, N. A. , & Reich, D. (2006). Principal components analysis corrects for stratification in genome‐wide association studies. Nature Genetics, 38, 904–909. 10.1038/ng1847 16862161

[ece34952-bib-0054] Pritchard, J. K. , Stephens, M. , & Donnelly, P. (2000). Inference of population structure using multilocus genotype data. Genetics, 155, 945–959. 10835412 10.1093/genetics/155.2.945 PMC1461096

[ece34952-bib-0055] R Core Team (2016). R: A language and environment for statistical computing. Vienna, Austria: R Foundation for Statistical Computing Retrieved from https://www.R-project.org/

[ece34952-bib-0056] Raj, A. , Stephens, M. , & Pritchard, J. K. (2014). fastSTRUCTURE: Variational inference of population structure in large SNP data sets. Genetics, 197, 573–589. 10.1534/genetics.114.164350 24700103 PMC4063916

[ece34952-bib-0057] Ramachandran, S. , Deshpande, O. , Roseman, C. C. , Rosenberg, N. A. , Feldman, M. W. , & Cavalli‐Sforza, L. L. (2005). Support from the relationship of genetic and geographic distance in human populations for a serial founder effect originating in Africa. Proceedings of the National Academy of Sciences, 102, 15942–15947. https://doi.org/10.1073pnas.0507611102 10.1073/pnas.0507611102 PMC1276087 16243969

[ece34952-bib-0058] Reitzel, A. M. , Herrera, S. , Layden, M. J. , Martindale, M. Q. , & Shank, T. M. (2013). Going where traditional markers have not gone before: Utility of and promise for RAD sequencing in marine invertebrate phylogeography and population genomics. Molecular Ecology, 22, 2953–2970. 10.1111/mec.12228 23473066 PMC3669247

[ece34952-bib-0059] Riedel, G. , Platt, B. , & Micheau, J. (2003). Glutamate receptor function in learning and memory. Behavioural Brain Research, 140, 1–47. 10.1016/S0166-4328(02)00272-3 12644276

[ece34952-bib-0060] Rius, M. , Bourne, S. , Hornsby, H. G. , & Chapman, M. A. (2015). Applications of next‐generation sequencing to the study of biological invasions. Current Zoology, 61, 488–504. 10.1093/czoolo/61.3.488

[ece34952-bib-0061] Schofield, P. J. (2009). Geographic extent and chronology of the invasion of non‐native lionfish (*Pterois volitans* [Linnaeus 1758] and *P. miles* [Bennett 1828]) in the Western North Atlantic and Caribbean Sea. Aquatic Invasions, 4, 473–479. 10.3391/ai.2009.4.3

[ece34952-bib-0062] Schofield, P. J. (2010). Update on geographic spread of invasive lionfishes (*Pterois volitans* [Linnaeus, 1758] and *P. miles* [Bennett, 1828]) in the Western North Atlantic Ocean, Caribbean Sea and Gulf of Mexico. Aquatic Invasions, 5, S117–S122. 10.3391/ai.2010.5.S1.024

[ece34952-bib-0063] Shum, P. , Pampoulie, C. , Kristinsson, K. , & Mariani, S. (2015). Three‐dimensional post‐glacial expansion and diversification of an exploited oceanic fish. Molecular Ecology, 24, 3652–3667. 10.1111/mec.13262 26073046 PMC4744735

[ece34952-bib-0064] Silva, G. , Horne, J. B. , & Castilho, R. (2014). Anchovies go north and west without losing diversity: Post‐glacial range expansions in a small pelagic fish. Journal of Biogeography, 41, 1171–1182. 10.1111/jbi.12275

[ece34952-bib-0065] Tan, W. , Aizen, J. , & Thomas, P. (2014). Membrane progestin receptor alpha mediates progestin‐induced sperm hypermotility and increased fertilization success in southern flounder (Paralichthys lethostigma). General and Comparative Endocrinology, 200, 18–26. 10.1016/j.ygcen.2014.02.003 24530629

[ece34952-bib-0066] Tepolt, C. K. , & Palumbi, S. R. (2015). Transcriptome sequencing reveals both neutral and adaptive genome dynamics in a marine invader. Molecular Ecology, 24, 4145–4158. 10.1111/mec.13294 26118396

[ece34952-bib-0067] Therkildsen, N. O. , Hemmer‐Hansen, J. , Als, T. D. , Swain, D. P. , Morgan, M. J. , Trippel, E. A. , … Nielsen, E. E. (2013). Microevolution in time and space: SNP analysis of historical DNA reveals dynamic signatures of selection in Atlantic cod. Molecular Ecology, 22, 2424–2440. 10.1111/mec.12260 23551301

[ece34952-bib-0068] Toledo‐Hernández, C. , Vélez‐Zuazo, X. , Ruiz‐Diaz, C. P. , Patricio, A. R. , Mege, P. , Navarro, M. , … Papa, R. (2014). Population ecology and genetics of the invasive lionfish in Puerto Rico. Aquatic Invasions, 9, 227–237. 10.3391/ai.2014.9.2.12

[ece34952-bib-0069] Tubbs, C. , Pace, M. , & Thomas, P. (2010). Expression and gonadotropin regulation of membrane progestin receptor alpha in Atlantic croaker (Micropogonias undulatus) gonads: Role in gamete maturation. General and Comparative Endocrinology, 165, 144–154. 10.1016/j.ygcen.2009.06.017 19539624

[ece34952-bib-0070] van Etten, J. (2015). gdistance: Distances and Routes on Geographical Grids R package version 1.1‐9. Retrieved from https://CRAN.R-project.org/package=gdistance

[ece34952-bib-0071] Vivanco, I. , & Sawyers, C. L. (2002). The phosphatidylinositol 3‐Kinase–AKT pathway in human cancer. Nature Reviews Cancer, 2, 489–501. 10.1038/nrc839 12094235

[ece34952-bib-0072] White, T. A. , Perkins, S. E. , Heckel, G. , & Searle, J. B. (2013). Adaptive evolution during an ongoing range expansion: The invasive bank vole (*Myodes glareolus*) in Ireland. Molecular Ecology, 22, 2971–2985. 10.1111/mec.12343 23701376

[ece34952-bib-0073] Wilcox, C. L. , Motomura, H. , Matsunuma, M. , & Bowen, B. W. (2018). Phylogeography lionfishes (*Pterois*) indicate taxonomic over splitting and hybrid origin of the invasive *Pterois volitans* . Journal of Heredity, 109(2), 162–175. 10.1093/jhered/esx056 28637254

[ece34952-bib-0074] Zhu, Y. , Rice, C. D. , Pang, Y. , Pace, M. , & Thomas, P. (2003). Cloning, expression, and characterization of a membrane progestin receptor and evidence it is an intermediary in meiotic maturation of fish oocytes. Proceedings of the National Academy of Sciences, 100, 2231–2236. 10.1073/pnas.0336132100 PMC151323 12574519

