# Supplementary material for: Population genomics of rapidly invading lionfish in the Caribbean reveals signals of range expansion in the absence of spatial population structure
Source: Ecol Evol. 2019 Feb 10;9(6):3306–20. doi: 10.1002/ece3.4952 (PMC6434604; doi:10.1002/ece3.4952)
Supplement: Supplementary file 1 [file ECE3-9-3306-s001.pdf]

SUPPLEMENTAL INFORMATION

Population Genomics of Rapidly Invading Lionfish in the Caribbean Reveals Signals of Range Expansion in the Absence of Spatial Population Structure

Appendix S1: Sample collection information and size distributions

Samples were collected from a variety of locations, habitat types, and depths within each region. Ages of fish used in RAD-sequencing were estimated using a growth curve as described in the methods and the likely year of recruitment was calculated from age (in days) and date of collection (Figure I.1). The locations used to calculate distances are presented in Table I.1. The sample location, length (standard and/or total), sex, weight, latitude and longitude of location collected, habitat type, collection date, and depth if available is presented in Table I.2 for all collected and used individuals.

Figure I.1. (A) Lionfish ages calculated from length measurements, and (B) likely recruitment years for samples included in the RAD-seq portion of the study.

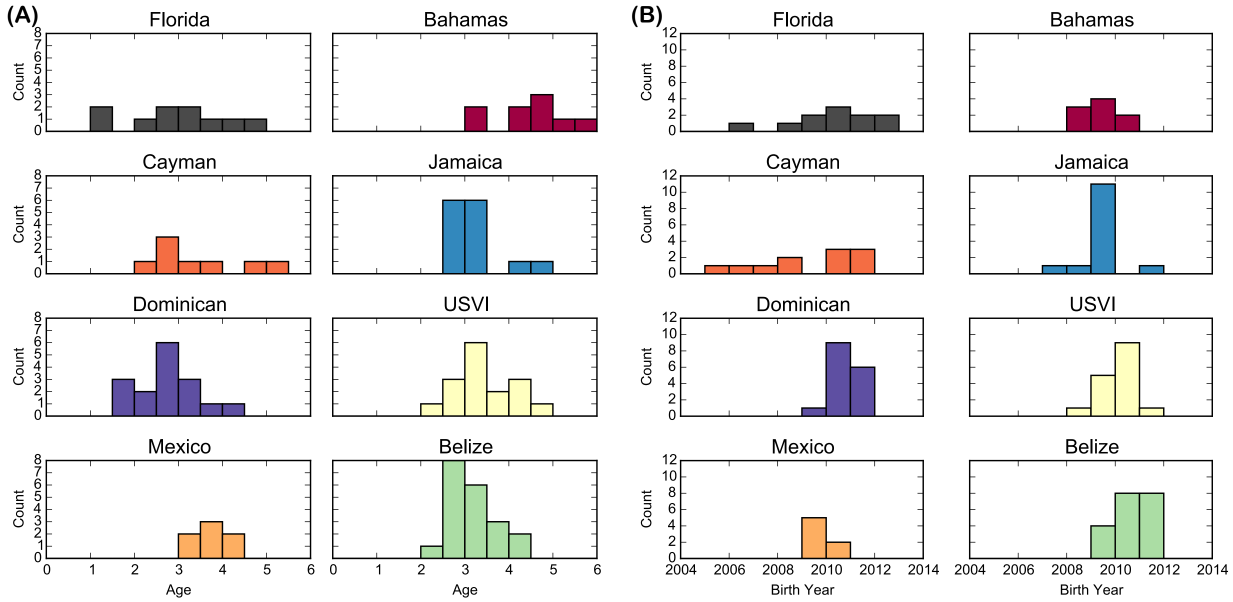

Table I.1. Latitude and longitude used in distance calculations (often the most common location of collection for individual fish).

Location	Dive site name	Latitude (N)	Longitude (W)
Florida, USA	Biscayne Bay	25.5662	-80.0906
The Bahamas	"Ron's Revenge"	24.5213	-76.2153
Jamaica	"English Reef"	17.8728	-77.7654
Dominican Republic	Bayahibe	18.3431	-68.8338
Grand Cayman	"Pedro's Castle"	19.2615	-81.2800
US Virgin Islands	Buck Island 1	17.7824	-64.6198
Mexico	Cozumel Marine Park	20.4547	-86.9922
Honduras	Roatan Island	16.2106	-86.3241
Belize	Ambergris Caye	18.1197	-87.8221

Table I.2. Sample information for each individual used in the RAD-sequencing portion of this study.

Sample	General Site	Collection Site Name	Standard	Total	Sex	Weight	Habitat type	Collection	Latitude	Longitude	Depth
EKB-445	Bahamas (2013)	barge	320.0	386.0	M	610.00	Coral Reef	10/23/2013	NA	NA	50
EKB-446	Bahamas (2013)	Ron's Revenge	278.0	346.0	M	627.00	Coral Reef	10/10/2013	24.521313	-76.215259	30
EKB-447	Bahamas (2013)	barge	280.0	370.0	M	581.00	Coral Reef	10/23/2013	NA	NA	50
EKB-451	Bahamas (2013)	Tunnel Rock	226	310.0	F	370.00	Coral Reef	10/2/2013	NA	NA	35
EKB-452	Bahamas (2013)	Ron's Revenge	281	362.0	M	479.00	Coral Reef	10/10/2013	24.521313	-76.215259	30
EKB-457	Bahamas (2013)	High rock	288.0	375.0	M	603.00	Coral Reef	10/16/2013	NA	NA	35
EKB-459	Bahamas (2013)	barge	289.0	358.0	M	548.00	Coral Reef	10/23/2013	NA	NA	50
EKB-460	Bahamas (2013)	barge	279.0	364.0	M	550.00	Coral Reef	10/23/2013	NA	NA	50
EKB-461	Bahamas (2013)	Random Reef	225.0	295.0	M	315.00	Coral Reef	10/10/2013	24.533505	-76.241298	15
EKB-489	Belize (2013)	East Snake, Port Honduras Marine Reserve	248.0	324.0	F	450.00	Coral Reef	8/9/13	16.207774	-88.508053	45
EKB-491	Belize (2013)	Chub Hole, Bacalar Chico Marine Reserve	240.0	310.0	M	442.00	Fringing Reef with many crevices	10/30/13	18.119658	-87.822099	60
EKB-492	Belize (2013)	Remora's Revenge, Caye Chapel	237.0	309.0	M	389.00	spur and groove	10/31/13	17.668380	-88.013850	66
EKB-493	Belize (2013)	Chub Hole, Bacalar Chico Marine Reserve	265.0	345.0	M	1000.00	spur and groove	10/26/13	18.155800	-87.821240	70
EKB-497	Belize (2013)	Remora's Revenge, Caye Chapel	189.0	246.0	F	173.00	spur and groove	10/31/13	17.668380	-88.013850	66
EKB-498	Belize (2013)	Remora's Revenge, Caye Chapel	210.0	280.0	F	190.00	spur and groove	10/31/13	17.668380	-88.013850	66
EKB-500	Belize (2013)	Sand Trap, Caye Chapel	205.0	264.0	M	197.00	spur and groove	10/31/13	17.691630	-88.008660	52
EKB-501	Belize (2013)	Sand Trap, Caye Chapel	211.0	278.5	M	232.00	spur and groove	10/31/13	17.691630	-88.008660	52
EKB-502	Belize (2013)	Chub Hole, Bacalar Chico Marine Reserve	270.0	350.0	M	604.00	Fringing Reef with many crevices	10/30/13	18.119658	-87.822099	60
EKB-503	Belize (2013)	Chub Hole, Bacalar Chico Marine Reserve	205.0	280.0	F	226.00	Fringing Reef with many crevices	10/30/13	18.119658	-87.822099	60
EKB-504	Belize (2013)	Chub Hole, Bacalar Chico Marine Reserve	225.0	280.0	F	337.00	Fringing Reef with many crevices	10/30/13	18.119658	-87.822099	60
EKB-528	Belize (2013)	Goliath, Bacalar Chico Marine Reserve	227.0	295.0	F	700.00	spur & groove	10/26/13	18.155800	-87.821240	70
EKB-612	Belize (2013)	Chub Hole, Bacalar Chico Marine Reserve	220.0	290.0	F	371.00	Fringing Reef with many crevices	10/30/13	18.119658	-87.822099	60
EKB-613	Belize (2013)	East Snake, PHMR, Honduras Marine Reserve, Belize	264.0	342.0	M	500.00	Coral Reef	8/9/13	16.207774	-88.508053	45
EKB-616	Belize (2013)	Chub Hole, Bacalar Chico Marine Reserve	210.0	280.0	M	455.00	Fringing Reef with many crevices	10/30/13	18.119658	-87.822099	70
EKB-617	Belize (2013)	Goliath, Bacalar Chico Marine Reserve	240.0	315.0	M	850.00	spur & groove	10/26/13	18.155800	-87.821240	70
EKB-618	Belize (2013)	Goliath, Bacalar Chico Marine Reserve	260.0	340.0	M	800.00	spur & groove	10/26/13	18.155800	-87.821240	70
EKB-619	Belize (2013)	Goliath, Bacalar Chico Marine Reserve	225.0	295.0	F	750.00	spur & groove	10/26/13	18.155800	-87.821240	70
EKB-621	Belize (2013)	Remora's Revenge, Caye Chapel	196.0	261.0	F	215.00	spur & groove	10/31/13	17.668380	-88.013850	66
EKB-624	Belize (2013)	Chub Hole, Bacalar Chico Marine Reserve	220.0	300.0	M	348.00	Fringing Reef with many crevices	10/30/13	18.119658	-87.822099	60
EKB-506	Cayman Islands (2013)	LC SPAG, Little Cayman	192.0	255.0	NA	250.00	wall patch	10/21/13	19.65190	80.10772	95
EKB-507	Cayman Islands (2013)	LC SPAG, Little Cayman	203.0	263.0	NA	250.00	wall patch	10/21/13	19.65190	80.10772	95
EKB-508	Cayman Islands (2013)	LC SPAG, Little Cayman	195.0	263.0	NA	250.00	wall patch	10/21/13	19.65190	80.10772	95
EKB-509	Cayman Islands (2013)	Spotts Beach Mooring, Grand Cayman	315.0	410.0	NA	850.00	Wall	10/12/13	19.26862	81.31138	75
EKB-510	Cayman Islands (2013)	Pedro Reef, Grand Cayman	303.0	--	NA	850.00	spur & groove	10/12/13	19.26657	81.29439	30
EKB-511	Cayman Islands (2013)	East of Black Forest, Grand Cayman	NA	282.0	NA	700.00	wall	10/12/13	19.27234	81.39530	80
EKB-512	Cayman Islands (2013)	Pedro Castle, Grand Cayman	287.0	370.0	NA	650.00	spur & groove	10/13/13	19.26148	81.28001	50
EKB-513	Cayman Islands (2013)	Pedro Castle, Grand Cayman	290.0	384.0	NA	800.00	spur & groove	10/13/13	19.26148	81.28001	50
EKB-514	Cayman Islands (2013)	Pilot Wrek, Grand Cayman	230.0	300.0	NA	350.00	wreck	10/13/13	19.36749	81.36927	10
EKB-515	Cayman Islands (2013)	NE Coast, Cayman Brac	318.0	412.0	NA	850.00	wall	10/21/13	19.74171	79.78287	95
EKB-516	Cayman Islands (2013)	NE Coast, Cayman Brac	238.0	326.0	NA	400.00	wall	10/21/13	19.74171	79.78287	95
EKB-529	Dominican Republic (2013)	La Caleta MPA	270.0	210.0	NA	246.40	Coral Reef	10/5/13	18.441100	69.694067°	80
EKB-530	Dominican Republic (2013)	La Caleta MPA	310.0	240.0	NA	336.90	Coral Reef	10/5/13	18.441100	69.694067°	80
EKB-531	Dominican Republic (2013)	SOSUA	260	350	NA	20onz	Coral Reef	10/20/13	19° 46.109'N	70° 33.688'W	80
EKB-532	Dominican Republic (2013)	La Caleta MPA	260.0	200.0	NA	210.10	Coral Reef	10/5/13	18.441539	69.693642°	80
EKB-533	Dominican Republic (2013)	La Caleta MPA	270.0	200.0	NA	244.70	Coral Reef	10/5/13	18.441539	69.693642°	80
EKB-534	Dominican Republic (2013)	BAYAHIBE	216.0	285.0	NA	275.00	Patch Reef	9/22/13	18.343100	68.833810	50
EKB-535	Dominican Republic (2013)	BAYAHIBE	224.0	288.0	NA	325.00	Patch Reef	9/22/13	18.343100	68.833810	50
EKB-536	Dominican Republic (2013)	Las Galeras Samana	320.0	240.0	NA	480.00	Coral Reef	10/12/13	19.17.474	069.11.902	108

EKB-537	Dominican Republic (2013)	Las Galeras Samana	370.0	290.0	NA	780.00	Coral Reef	10/12/13	19.17.474	069.11.902	108
EKB-538	Dominican Republic (2013)	Las Galeras Samana	330.0	260.0	NA	600.00	Coral Reef	10/12/13	19.17.474	069.11.902	108
EKB-605	Dominican Republic (2013)	SOSUA	230.0	310.0	NA	10onz	Coral Reef	10/20/13	19° 46.109'N	70° 33.688'W	80
EKB-607	Dominican Republic (2013)	SOSUA	260.0	330.0	NA	11onz	Coral Reef	10/20/13	19° 45.519'N	70° 31.230'	100
EKB-608	Dominican Republic (2013)	BAYAHIBE	215.0	282.0	NA	220.00	Patch Reef	9/22/13	18.343100	68.833810	50
EKB-609	Dominican Republic (2013)	La Caleta MPA	400.0	310.0	NA	450.3	Coral Reef	10/5/13	18.441539	69.693642°	80
EKB-610	Dominican Republic (2013)	BAYAHIBE	217.0	285.0	NA	300.00	Patch reef	9/22/13	18.343100	68.833810	50
EKB-611	Dominican Republic (2013)	BAYAHIBE	232.0	297.0	NA	390.00	Patch reef	9/22/13	18.343100	68.833810	50
EKB_026	Biscayne Bay, FL	Bluefire	315	NA	M		Wreck	8/29/2013	25°33'58.56"N	80° 5'26.28"W	110-120
EKB_030	Biscayne Bay, FL	Bluefire	265	NA	M		Wreck	8/29/2013	25°33'58.56"N	80° 5'26.28"W	110-120
EKB_031	Biscayne Bay, FL	Bluefire	229	NA	M		Wreck	8/29/2013	25°33'58.56"N	80° 5'26.28"W	110-120
EKB_032	Biscayne Bay, FL	Bluefire	235	NA	M		Wreck	8/29/2013	25°33'58.56"N	80° 5'26.28"W	110-120
EKB_033	Biscayne Bay, FL	Bluefire	222	NA	F		Wreck	8/29/2013	25°33'58.56"N	80° 5'26.28"W	110-120
EKB_034	Biscayne Bay, FL	Bluefire	199	NA	M		Wreck	8/29/2013	25°33'58.56"N	80° 5'26.28"W	110-120
EKB_035	Biscayne Bay, FL	Bluefire	184	NA	M		Wreck	8/29/2013	25°33'58.56"N	80° 5'26.28"W	110-120
EKB_036	Biscayne Bay, FL	Bluefire	103	NA	U		Wreck	8/29/2013	25°33'58.56"N	80° 5'26.28"W	110-120
EKB_037	Biscayne Bay, FL	Bluefire	99	NA	U		Wreck	8/29/2013	25°33'58.56"N	80° 5'26.28"W	110-120
EKB_038	Biscayne Bay, FL	Bluefire	257	NA	U		Wreck	8/29/2013	25°33'58.56"N	80° 5'26.28"W	110-120
EKB_040	Biscayne Bay, FL	Long Reef (Drift Dive)	285	NA	M		Deep Reef	8/29/2013	25°26.620' N	80°06.912' W	70-80
EKB-421	Honduras (2013)	Roatan Island, Odessey Wreck	NA	NA	U		Wreck / Artificial Reef	10/31/2013	16.2105690	-86.3240890	140
EKB-422	Honduras (2013)	Roatan Island, Odessey Wreck	NA	NA	U		Wreck / Artificial Reef	10/31/2013	16.2105690	-86.3240890	140
EKB-423	Honduras (2013)	Roatan Island, Odessey Wreck	NA	NA	U		Wreck / Artificial Reef	10/31/2013	16.2105690	-86.3240890	140
EKB-424	Honduras (2013)	Roatan Island, Odessey Wreck	NA	NA	U		Wreck / Artificial Reef	10/31/2013	16.2105690	-86.3240890	140
EKB-426	Honduras (2013)	Roatan Island, Odessey Wreck	NA	NA	U		Wreck / Artificial Reef	10/31/2013	16.2105690	-86.3240890	140
EKB-427	Honduras (2013)	Roatan Island, Odessey Wreck	NA	NA	U		Wreck / Artificial Reef	10/31/2013	16.2105690	-86.3240890	140
EKB-429	Honduras (2013)	Roatan Island, Odessey Wreck	NA	NA	U		Wreck / Artificial Reef	10/31/2013	16.2105690	-86.3240890	140
EKB-431	Honduras (2013)	Roatan Island, Odessey Wreck	NA	NA	U		Wreck / Artificial Reef	10/31/2013	16.2105690	-86.3240890	140
EKB-433	Honduras (2013)	Roatan Island, Odessey Wreck	NA	NA	U		Wreck / Artificial Reef	10/31/2013	16.2105690	-86.3240890	140
EKB-434	Honduras (2013)	Roatan Island, Odessey Wreck	NA	NA	U		Wreck / Artificial Reef	10/31/2013	16.2105690	-86.3240890	140
EKB-436	Honduras (2013)	Mangrove Bight, Guanaja Island	NA	NA	U		Coral Reef	10/23/2013	16.313200	-85.525100	40
EKB-438	Honduras (2013)	Mangrove Bight, Guanaja Island	NA	NA	U		Coral Reef	10/23/2013	16.313200	-85.525100	40
EKB-439	Honduras (2013)	Mangrove Bight, Guanaja Island	NA	NA	U		Coral Reef	10/23/2013	16.313200	-85.525100	40
EKB-441	Honduras (2013)	Mangrove Bight, Guanaja Island	NA	NA	U		Coral Reef	10/23/2013	16.313200	-85.525100	40
EKB-442	Honduras (2013)	Mangrove Bight, Guanaja Island	NA	NA	U		Coral Reef	10/23/2013	16.313200	-85.525100	40
EKB-443	Honduras (2013)	Mangrove Bight, Guanaja Island	NA	NA	U		Coral Reef	10/23/2013	16.313200	-85.525100	40
EKB-464	Jamaica (2012)	Dairy Bull	222	296	M	372	Coral Reef	7/17/12	18.473376	-77.387518	NA
EKB-468	Jamaica (2012)	East Fore Reef	267	353	M	464	Coral Reef	7/13/12	NA	NA	NA
EKB-471	Jamaica (2012)	English Reef	221	294	M	290	Coral Reef	7/29/12	NA	NA	88
EKB-472	Jamaica (2012)	Fish Pot (Bluefields)	194	265	F	500	Coral Reef	7/28/12	NA	NA	NA
EKB-473	Jamaica (2012)	Fish Pot (Bluefields)	205	272	F	500	Coral Reef	7/28/12	NA	NA	NA
EKB-474	Jamaica (2012)	Fish Pot (Bluefields)	202	281	M	550	Coral Reef	7/26/12	NA	NA	NA
EKB-475	Jamaica (2012)	English Reef	206	269	F	250	Coral Reef	7/29/12	NA	NA	88
EKB-477	Jamaica (2012)	English Reef	228	307	M	450	Coral Reef	7/29/12	NA	NA	88
EKB-478	Jamaica (2012)	English Reef	226	306	M	310	Coral Reef	7/29/12	NA	NA	88
EKB-479	Jamaica (2012)	East Fore Reef	227	305	M	350	Coral Reef	7/13/14	NA	NA	NA
EKB-480	Jamaica (2012)	Fish Pot (Bluefields)	267	364	M	850	Coral Reef	7/26/12	NA	NA	NA
EKB-483	Jamaica (2012)	Dairy Bull	231	311	M	390	Coral Reef	7/17/12	NA	NA	80
EKB-484	Jamaica (2012)	English Reef	212	284	M	300	Coral Reef	7/29/12	NA	NA	88
EKB-485	Jamaica (2012)	Fish Pot (Bluefields)	195	265	F	500	Coral Reef	7/26/12	NA	NA	NA

EKB-406	Mexico (2013)	Cozumel Marine Park	220.0	302.0	m		Hard Bottom	8/25/2013	20.4546540	-86.99218	100
EKB-407	Mexico (2013)	Cozumel Marine Park	255.0	350.0	m		Hard Bottom	8/25/2013	20.4546540	-86.99218	100
EKB-410	Mexico (2013)	Cozumel Marine Park	258.0	346.0	m		Hard Bottom	8/25/2013	20.4546540	-86.99218	100
EKB-412	Mexico (2013)	Cozumel Marine Park	255.0	340.0	m		Hard Bottom	8/25/2013	20.4546540	-86.99218	100
EKB-415	Mexico (2013)	Cozumel Marine Park	260.0		m		Hard Bottom	8/25/2013	20.4546540	-86.99218	100
EKB-416	Mexico (2013)	Cozumel Marine Park	242.0	312.0	f		Hard Bottom	8/25/2013	20.4546540	-86.99218	100
EKB-419	Mexico (2013)	Cozumel Marine Park	250.0	335.0	m		Hard Bottom	8/25/2013	20.4546540	-86.99218	100
EKB-361	USVI (2013)	P3-8	195	NA	M		Patch Reef	2/17/2013	17.78228	-64.60800	
EKB-362	USVI (2013)	LF5-4	264	NA	F		Continuous Reef	5/12/2013	17.80367	-64.63655	
EKB-364	USVI (2013)	LF4-10	245	NA	M		Continuous Reef	2/17/2013	17.79705	-64.63998	
EKB-365	USVI (2013)	I2-5	239	NA	F		Fringing Reef	5/11/2013	17.78340	-64.61470	
EKB-367	USVI (2013)	I2-6	271	NA	F		Fringing Reef	5/11/2013	17.78340	-64.61470	
EKB-368	USVI (2013)	I1-1	219	NA	M		Fringing Reef	5/12/2013	17.78241	-64.61976	
EKB-369	USVI (2013)	P3-1	267	NA	F		Patch Reef	5/11/2013	17.78228	-64.60800	
EKB-373	USVI (2013)	I1-1	203	NA	F		Fringing Reef	10/26/2013	17.78241	-64.61976	
EKB-374	USVI (2013)	LF5-3	286	NA	M		Continuous Reef	8/25/2013	17.80367	-64.63655	
EKB-375	USVI (2013)	P5-1	244	NA	M		Patch Reef	8/24/2013	17.77763	-64.59573	
EKB-378	USVI (2013)	LF5-1	198	NA	U		Continuous Reef	6/22/2013	17.80367	-64.63655	
EKB-379	USVI (2013)	P3-3	242	NA	M		Patch Reef	10/26/2013	17.78228	-64.60800	
EKB-380	USVI (2013)	I2-1	241	NA	M		Fringing Reef	8/24/2013	17.78340	-64.61470	
EKB-381	USVI (2013)	LF5-1	229	NA	M		Continuous Reef	8/25/2013	17.80367	-64.63655	
EKB-382	USVI (2013)	P3-1	246	NA	M		Patch Reef	10/26/2013	17.78228	-64.60800	
EKB-383	USVI (2013)	P1-1	250	NA	I		Patch Reef	6/22/2013	17.77933	-64.61403	

Appendix S2: Quality and quantity of RAD-sequencing in the *Stacks* pipeline

Process_radtags program specifics

For the single-end sequence plate (95 samples), Illumina sequencing of the prepared RAD libraries yielded 179,873,518 million reads, 1,238,285 (0.69%) of which were discarded due to low quality, 17,646,010 (9.81%) of which were discarded due to absence of a barcode, and 38,589,757 (21.45%) of which were discarded due to the absence of or ambiguity in the restriction site. After filtering, 122,399,466 (68.05%) were used moving forward in the *Stacks* pipeline (Figure II.1A). For the paired-end RAD-seq plate (25 samples for this study), only the first read of the paired end data are used here as population genomic data. Illumina sequencing of the prepared paired-end RAD libraries yielded 126,382,552 reads, 275,853 (0.22%) of which were discarded due to low quality, 20,527,070 (16.24%) were discarded due to absence of a barcode, and 26,373,050 (20.87%) were discarded due to the absence of or ambiguity in the restriction site. After filtering, 79,206,579 (62.67%) of the total reads were used moving forward in the *Stacks* pipeline (Figure II.1B). For single end Illumina sequencing, for each population, the percentage of reads discarded due to low quality was less than 0.5% and the percentage of reads discarded due to an ambiguous RAD-tag was between 18.2% (for the USVI samples) and 35.5% (for the Mexico samples). For the paired-end Illumina sequencing, for each population, the percentage of reads discarded due to low quality was less than 0.3% and the percentage discarded due to ambiguous barcodes ranged from 18.68% (for The Bahamas samples) and 29.54% (for the Honduras samples).

Process_radtags was run with *Stacks v.1.19* and the downstream analyses used *Stacks v.1.35.* While interoperability of the *.tags.tsv, *.snps.tsv, and *.matches.tsv were affected in the v.*1.20* update, the files generated by *process_radtags* were not affected and therefore could be used in *v.1.35* (for a summary of updates, see http://catchenlab.life.illinois.edu/stacks/).

Figure II.1: Total percentages of reads retained and filtered out due to ambiguous barcodes, ambiguous rad tags, or low quality by *process_radtags.* (A) for single end data, (B) for paired end data.

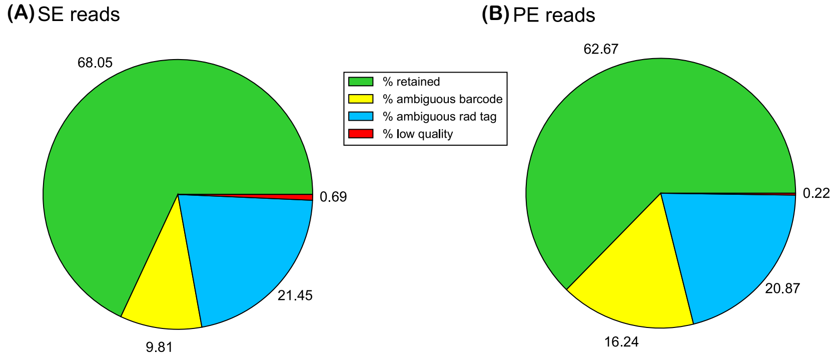

Denovomap.pl and populations programs results

The mean merged depth of coverage remained steady as more reads were added to the analysis, indicating that as reads were added, so were loci (Figure II.2). The sequencing type did not affect the ultimate outcome of depth of coverage, but individuals sequenced with paired-end Illumina sequencing had slightly higher total utilized reads (Figure II.2).

Figure II.2. Mean merged depth of coverage vs. number of utilized reads for each sample shown by population (top panel) and by sequencing type (bottom panel).

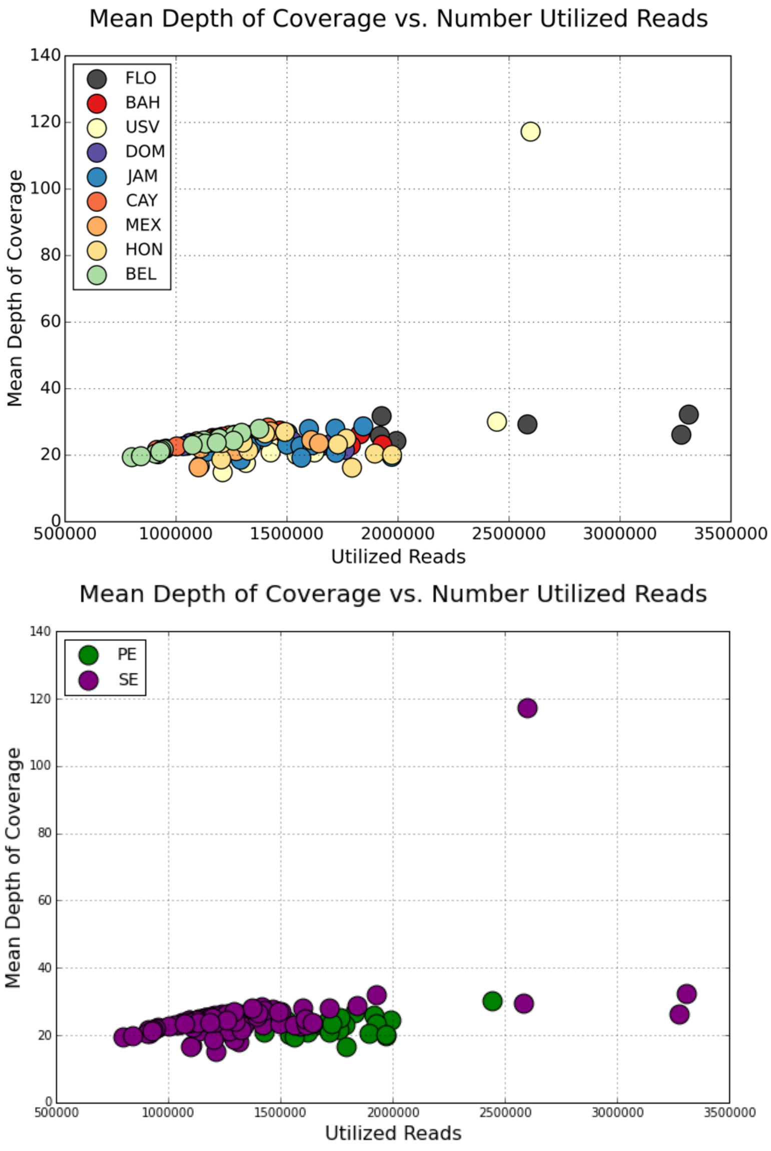

The filtering requirements by *Stacks populations* programs affected the number of loci used in analysis in predictable ways: the more stringent the requirements for loci being shared between

populations and individuals, the fewer loci were retained for analysis. The two ways to alter these requirements are with the –p and –r arguments in the program. As the requirements for the number of populations (-p) and percent of individuals within a population (-r) are relaxed, the number of loci used converged on a range between 40,000-50,000 with 25,000 to 30,000 of those being variant loci that informed population genetic statistics (Figure II.3). When lower –r values (-r 0.7) were run through downstream analyses, the same trends in population genetic summary statistics were observed as those reported in this paper. That said, if –r is reduced to 0.5, then in some cases only 4 or 5 individuals would be required by Stacks to use the individual. An *n* of 4 or 5 is too low for confidence in the population genetic analysis.

Figure II.3. Number of (A) total loci and (B) variant loci analyzed by the *populations* program in *STACKS* for different –p and –r parameters.

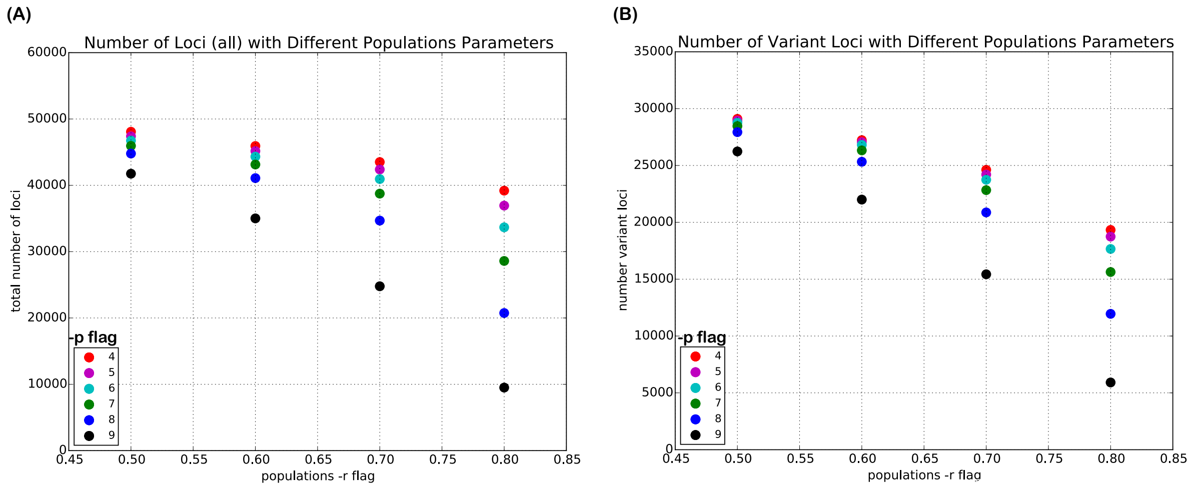

Appendix S3: Calculations of distance and impact of distance measurements on summary statistics regressions

As described in the Methods section, distance was calculated multiple ways (Table III.1), and summary statistics were regressed against each measurement (Figure III.1). Results for the regressions varied slightly with the "modified-ocean-distance" the best fit regression for the data (Table III.2).

Table III.1. Different calculations of distance (in km) from Florida using (1) Euclidian distance, (2) least-cost distance through the ocean with the only requirement being that the path remain in the water, and (3) least-cost distance through the ocean with the requirement that for sites to the West of Cuba, the path travel around the East side of the island.

Site	Euclidian	Ocean	Modified Ocean
Florida	0	0	0
Bahamas	406	409.02	409.02
Jamaica	885	1303.79	1303.79
Cayman Islands	678	1084.90	1556.56
Dominican Republic	1409	1518.20	1518.20
Mexico	906	889.1	2122.87
Belize	1148	1127.80	2239.69
Honduras	1222	1266.5	2180.68
US Virgin Islands	1816	1816.40	1816.40

Figure III.1. Regressions of observed heterozygosity against Euclidian distance and through-ocean distance measures. The modified ocean distance regression is in Figure 2 of the main chapter text.

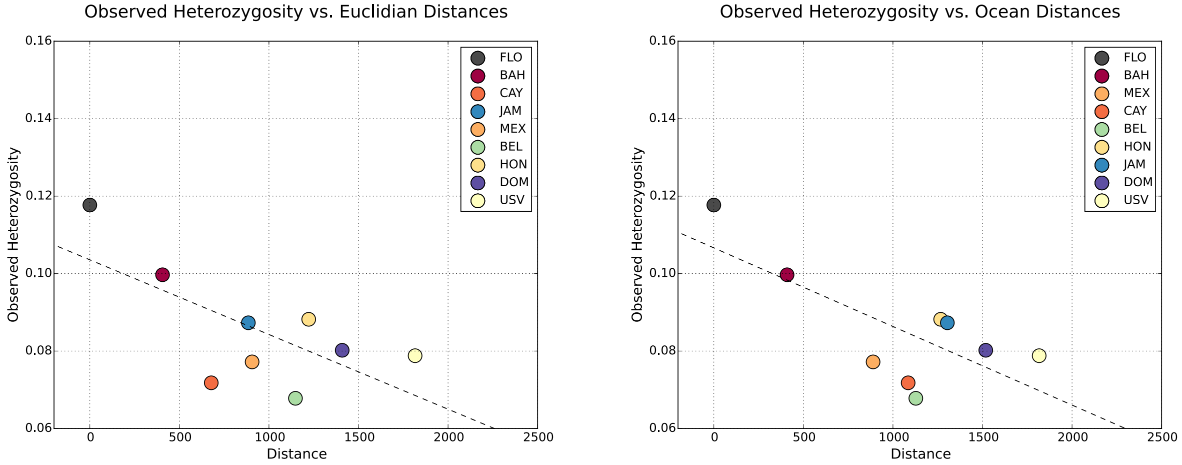

Table III.2. Regression results for observed heterozygosity for multiple distance-regimes.

**Distance Regime**	**R^2**	**p-value**
Euclidian	0.4623	0.044
Ocean	0.5365	0.025
Modified-Ocean	0.7436	0.003

Appendix S4. Results from mitochondrial D-Loop sequencing and analysis

Five haplotypes were sequenced across the nine study sites (Figure IV.1), corresponding to previously reported haplotypes named H01, H02, H03, H04, and H06 (Betancur-R *et al.* 2009). In previous research, nine haplotypes have been described in the entire invaded range, but only four of those have been described in the southern part of the expansion which is the focus of this study (not including The Bahamas, which is often considered to be part of the northern expansion) (Toledo-Hernández *et al*. 2014; Butterfield *et al*. 2015; Johnson *et al*. 2016). H02 is the most common haplotype, both in the present study (n = 146), and in previous studies (*e.g*., Butterfield *et al*. 2015). Haplotype H01 was sequenced in 61 individuals. H03, H04, and H06 were sequenced in 2, 7, and 1 individual(s) respectively.

Only four of the nine total haplotypes have been reported in the Caribbean (Betancur-R *et al.* 2011; Toledo-Hernández *et al.* 2014; Butterfield *et al.* 2015; Johnson *et al.* 2016). Most recently, the expansion into the Gulf of Mexico has resulted in a bottleneck between Caribbean populations and the Gulf of Mexico populations, evidenced by the existence of only three of the four haplotypes found in the Caribbean region (Johnson *et al.* 2016).

The four haplotypes that have ever been found south of The Bahamas (in this study and previous studies) are H01, H02, H03, and H04. Contrary to previous findings, haplotype H03 was not found in this study. However, this is not wholly unexpected because in previous studies, H03 was only observed in the Cayman Islands, Puerto Rico, and Panama. Puerto Rico and Panama are not included in this study, and the sample size from the Cayman Islands may be too small to detect a rare haplotype. Therefore, the results of this portion of the study are similar to what has been reported previously. The only exception to expected trends is the low haplotype diversity reported for Florida, where only two haplotypes were sequenced in this study.

The lower haplotype diversity in Florida differs from previous reports, in that previous papers have reported all 9 haplotypes in that area. One possible explanation is that all our samples are from a very small geographic area in Florida. Interestingly, the lower haplotype diversity of mtDNA was not mirrored in the RAD-seq data. In some previous papers (Butterfield *et al*. 2015), there has been discussion of possible second introductions or invasion origins in The Bahamas, but as there have been no new mitochondrial haplotypes discovered in the invaded range since the initial genetic work was published by Freshwater *et al*. (2009), we do not see evidence for multiple introductions.

Figure IV.1. Distribution of the mtDNA D-Loop haplotypes in the study area.

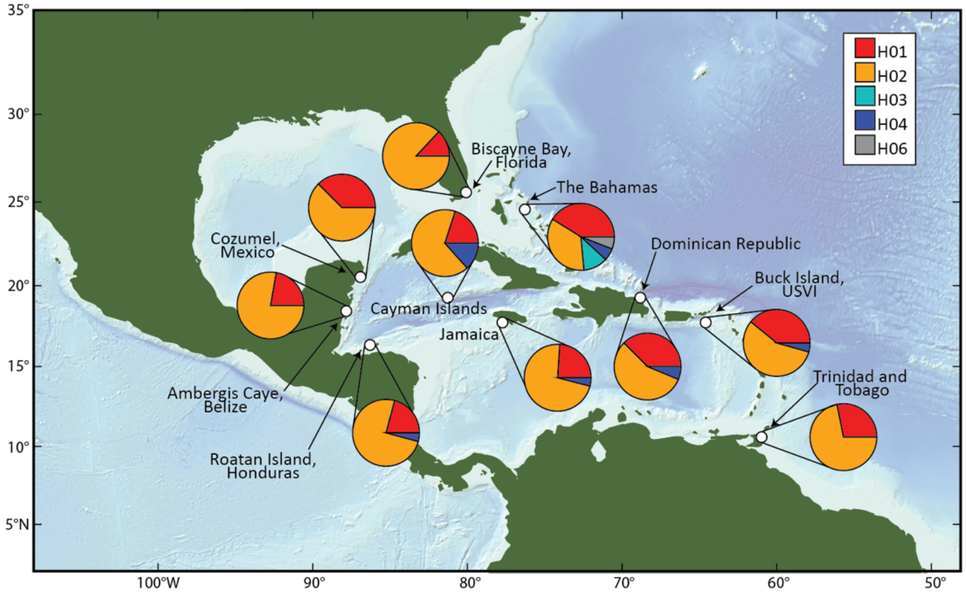

Appendix S5. Genome size estimates

Table SI.V.1. Cut site and genome size estimates as generated by PredRAD.

No. of Cut Sites	Lower estimate of genome size	Upper estimate of genome size
15,000	370,725,631	477,646,515
20,000	452,612,181	583,149,943
25,000	528,391,137	680,784,288

Appendix S6. Site Frequency Spectra and Sorted Allele F_{IS}

Figure VI.1. Site frequency spectra for each population showing the proportion of loci in each frequency bin (number of bins = 20) for the major allele (*p* as calculated by *Stacks*).

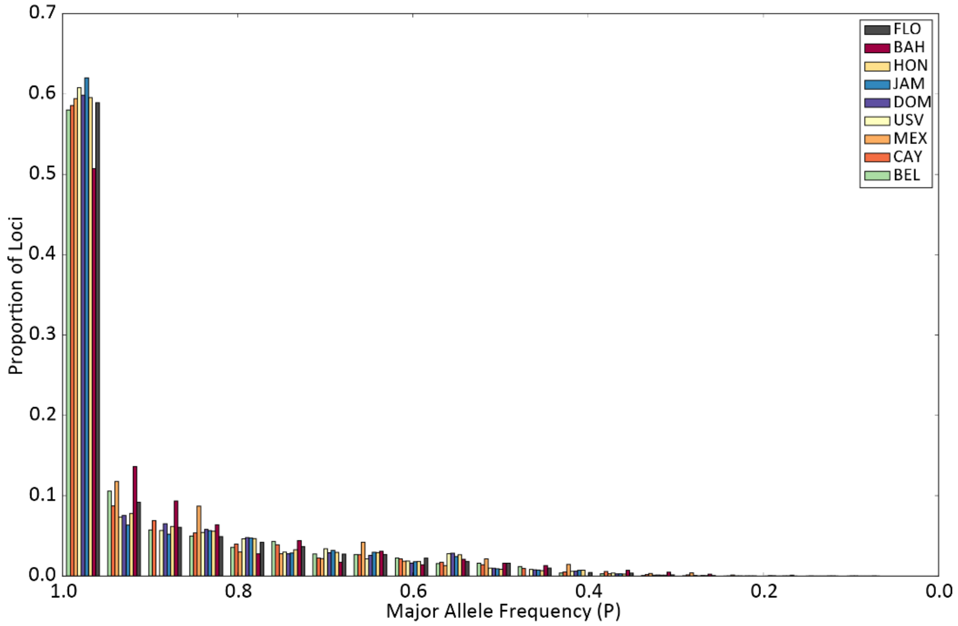

Figure VI.2. Site frequency spectra for different loci filtering methods showing the proportion of loci in each frequency bin (number of bins = 20) for the major allele (*p* as calculated by *Stacks*). The "flip flop" loci include all those that oscillated from below 0.5 to above 0.5, not just the ones with "great distance" (n = 1207).

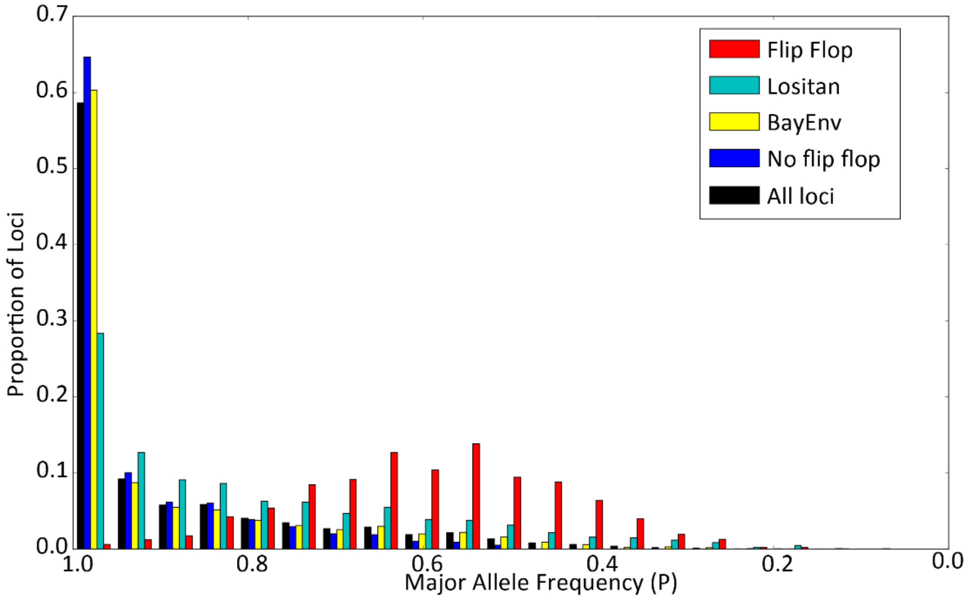

Figure SI.VI.3. F_{IS} distributions showing the proportion of loci with F_{IS} values for different filtering methods. Values were corrected as described in the Methods section. The "flip flop" loci include all those that oscillated from below 0.5 to above 0.5, not just the ones with "great distance" (n = 1207).

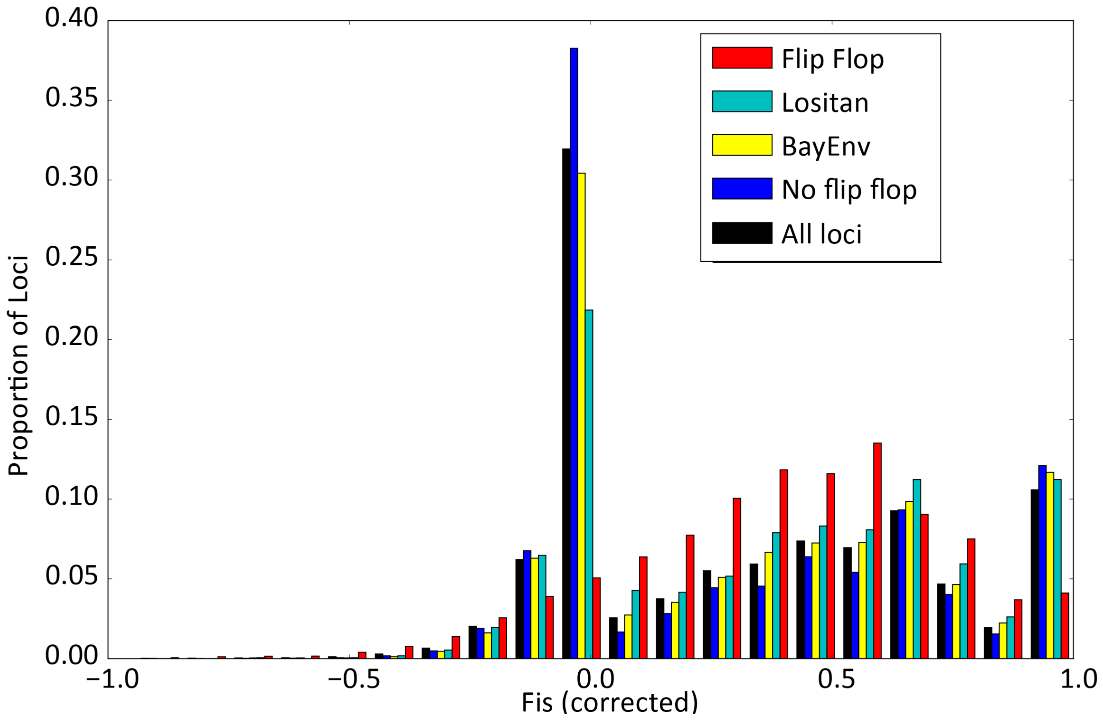

Appendix S7. BLAST Results

The identity of locus 48803, a putative glutamate receptor, was highly supported with no gaps in the alignment and between 93% and 95% identity matches with glutamate receptor sequences for the Asian sea bass, *Lates calcarifer*; the bicolor damselfish, *Stegastes partitus*; and the turquoise killifish, *nethobranchius furzeri*. The conserved domain analysis resulted in the identification of this gene region as being a periplasmic binding protein, type I, which is consistent with glutamate receptor proteins. For locus 11751, putatively identified by Blast2GO as a progestin receptor, the BLAST-n alignment yielded a maximum identity of 90% for a progestin receptor sequence from the Asian sea bass, *Lates calcarifer*. BLAST-nr results were the same for both loci. There was no conserved domain identified for locus 11751. Locus 54375, a potential antigen-like protein, was identified as part of the CLECT conserved domain, which includes c-type lectin-like protein domains found across a broad range of proteins, including those found in human dendritic cells and some antigen-like proteins. This locus was not able to be more specifically identified. Finally, there was high support for locus 15012 as a tyrosine kinase with a max identify score of 96%.

LITERATURE CITED IN THE SUPPLEMENTAL INFORMATION

Betancur-R R, Hines A, Acero P A *et al.* (2011) Reconstructing the lionfish invasion: insights into Greater Caribbean biogeography. *Journal of Biogeography*, **38**, 1281–1293.

Butterfield JSS, Díaz-Ferguson E, Silliman BR *et al.* (2015) Wide-ranging phylogeographic structure of invasive red lionfish in the Western Atlantic and Greater Caribbean. *Marine Biology*, **162**, 773–781.

Johnson J, Bird CE, Johnston MA, Fogg AQ, Hogan JD (2016) Regional genetic structure and genetic founder effects in the invasive lionfish: comparing the Gulf of Mexico, Caribbean and North Atlantic. *Marine Biology*, **163**, 1–7.

Toledo-Hernández C, Vélez-Zuazo X, Ruiz-Diaz CP *et al.* (2014) Population ecology and genetics of the invasive lionfish in Puerto Rico. *Aquatic Invasions*, **9**, 227–237.
